# Practical Synthesis and Application of Halogen-Doped Pyrrole Building Blocks

**DOI:** 10.1021/acsomega.1c00331

**Published:** 2021-03-30

**Authors:** Andrej Emanuel Cotman, Thomas Guérin, Ivana Kovačević, Davide Benedetto Tiz, Martina Durcik, Federica Fulgheri, Štefan Možina, Daniela Secci, Maša Sterle, Janez Ilaš, Anamarija Zega, Nace Zidar, Lucija Peterlin Mašič, Tihomir Tomašič, Frédéric R. Leroux, Gilles Hanquet, Danijel Kikelj

**Affiliations:** † Faculty of Pharmacy, University of Ljubljana, Aškerčeva cesta 7, 1000 Ljubljana, Slovenia; ‡ Université de Strasbourg, CNRS, UMR 7042-LIMA, ECPM, 25 Rue Becquerel, Strasbourg 67087, France; § Faculty of Sciences, Department of Chemistry, Biochemistry and Environmental Protection, University of Novi Sad, Trg Dositeja Obradovića 3, 21000 Novi Sad, Serbia

## Abstract

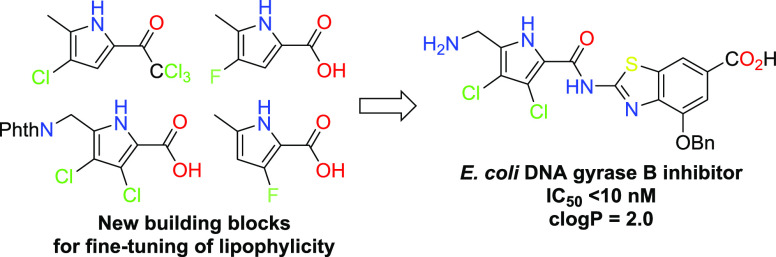

A practical access to four new halogen-substituted pyrrole building blocks was realized in two to five synthetic steps from commercially available starting materials. The target compounds were prepared on a 50 mg to 1 g scale, and their conversion to nanomolar inhibitors of bacterial DNA gyrase B was demonstrated for three of the prepared building blocks to showcase the usefulness of such chemical motifs in medicinal chemistry.

## 1 Introduction

**Figure 1:**
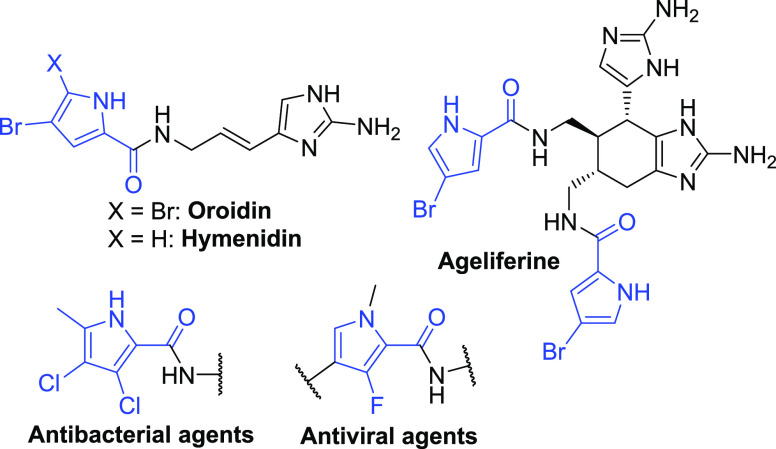
Representative natural products and bioactive compounds with a halogen-doped 1*H*-pyrrole-2-carboxamide fragment.

Halogen-substituted pyrrole-2-carboxamide is an integral molecular fragment of bioactive marine natural products as well as natural and synthetic anti-infectives (Figure 1). In particular, mono- and dibromopyrrole-2-carboxamide are found in oroidin^1^ and hymenidin,^2^ which are postulated precursors for structurally diverse mono- and oligomeric secondary metabolites involved in the chemical defense of *Agelas* marine sponges. A representative compound ageliferin^3^ features a complex multichiral scaffold.^4^ Furthermore, 3,4-dichloro-5-methyl-1*H*-pyrrole-2-carboxamide is a molecular fragment of natural^5,6^ and synthetic^7^ antibacterials, crucial for binding to the active site of bacterial topoisomerases, and the 3-fluoro-1*H*-pyrrole-2-carboxamide moiety is found in promising preclinical candidates, active against hepatitis B virus (Figure 1).^8^

**Figure 2:**
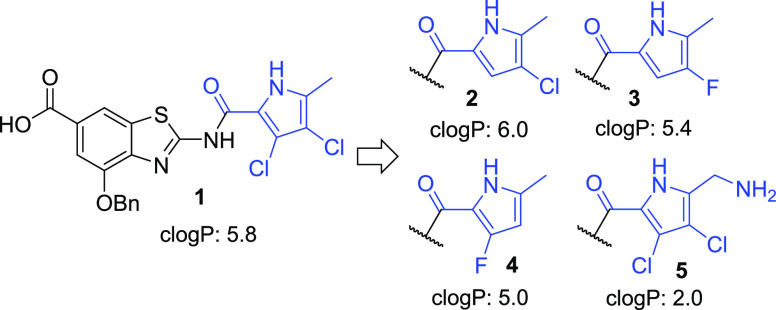
Design of antibacterial hit compound analogues with decreased lipophilicity.

During our ongoing research in the field of dual bacterial DNA gyrase/topoisomerase IV inhibitors,^9−15^ a promising hit compound **1** (Figure 2) was identified, displaying low nanomolar inhibition of the target enzymes and broad-spectrum activity against gram-positive bacterial strains.^16^ Due to the compound’s high lipophilicity, its more polar analogues **2**–**5** were designed by varying the 3,4-dichloro-5-methyl-1*H*-pyrrole moiety, envisioning improved physical properties (*c* log *P* was calculated by ChemDraw) of the analogues while retaining the on-target activity (Figure 2).

With no preceding literature on the synthesis of the required pyrrole building blocks for the preparation of compounds **2**–**5**, we report herein our synthetic endeavors, where the main goal was the timely delivery of at least 100 mg of the sample to be built into the bioactive molecules. The amide bond of the target compounds can be formed using pyrrole-2-carbonyl chloride or 2-trichloroacetylpyrrole; therefore, either would be an acceptable option.

## 2 Results and Discussion

**Scheme 1:**
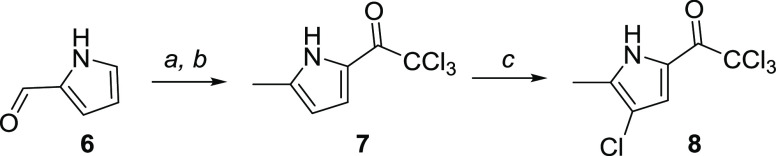
Synthesis of 2-Trichloroacetyl-4-chloro-5-methyl-1*H*-pyrrole **8** Reagents and conditions: (a) NH_2_NH_2_·H_2_O, ethylene glycol, 90 °C, 1 h, then KOH, 90 °C, 2.5 h (70% yield); (b) CCl_3_COCl, Et_2_O, r.t., 2 h (60% yield); and (c) *N*-chlorosuccinimide, dichloromethane, r.t., 4 h (61% yield).

The literature procedure for the synthesis of 4-chloro-5-methyl-1*H*-pyrrole-2-carboxylic acid involves chlorination of ethyl 5-methyl-1*H*-pyrrole-2-carboxylate using *N*-chlorosuccinimide at 0 °C and required in our hands laborious chromatographic separation of two barely resolved products.^17^ The practical synthesis of an alternative acylating agent **8** for the introduction of the same structural fragment was thus developed (Scheme 1). Trichloroacetylpyrrole **7** was prepared from pyrrole-2-carbaldehyde **6** employing the Wolff–Kishner reduction and Friedel–Crafts acylation.^18^ It was then directly monochlorinated using *N*-chlorosuccinimide at r.t. and the pure product **8** was obtained on the gram scale in 61% isolated yield after convenient crystallization from dichloromethane. Its structure was unambiguously determined by two-dimensional (2D) nuclear magnetic resonance (NMR) experiments (Supporting Information), showing that the electrophilic chlorination was selective for the position next to the electron-donating methyl substituent.

**Scheme 2:**
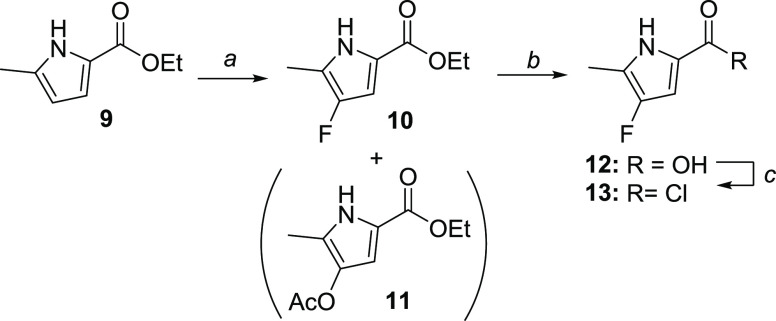
Synthesis of 4-Fluoro-5-methyl-1*H*-pyrrole-2-carbonyl Chloride **13** Reagents and conditions: (a) Selectfluor, MeCN/AcOH 5:1, 0 °C, 2 h (6.5% yield); (b) 10 M NaOH (aq), EtOH, 90 °C, 3 h (76% yield); and (c) oxalyl chloride, dichloromethane, r.t., overnight (quant. yield).

Next, we targeted the 4-fluoro-substituted building block. The screening of different halogen exchange (“Halex”) conditions involving crown ether 18-C-6 and [2.2.2]cryptand, for the conversion of chloropyrrole **8** or ethyl 4-chloro-5-methylpyrrole-2-carboxylate to the corresponding arylfluorides, returned no hits.^19^ We thus resorted to electrophilic fluorination of ethyl 5-methyl-1*H*-pyrrole-2-carboxylate **9** (Scheme 2). Initial 0.5 mmol scale screening of the reaction conditions (Table S1 in the Supporting Information) revealed that Selectfluor-mediated fluorination^20^ outperformed the *N*-fluorobenzenesulfonimide (NFSI)-mediated Lewis acid-catalyzed fluorination,^21^ as the former resulted in somewhat cleaner conversions. When the fluorination was performed at 0 °C in a mixture of acetonitrile and acetic acid (Table S2, entries 11 and 12), the formation of target compound **10**, accompanied by an acetoxy side product **11**, was observed. Their structures were confirmed by single-crystal X-ray diffraction analysis (Figures S1 and S2 in the Supporting Information). Aiming for an efficient med–chem synthetic route, the reaction was performed on a 2 g scale (Scheme 2), delivering **10** in a consistent 4.5–6.5% yield after flash chromatography. Ester **10** was hydrolyzed to acid **12**, requiring rather forcing conditions, and acyl chloride **13** was finally formed using oxalyl chloride in dichloromethane. It is noteworthy that acyl chloride formation using refluxing sulfonyl chloride or oxalyl chloride with the catalytic quantity of dimethylformamide (DMF) resulted in the formation of a significant amount of unidentified side products.

**Scheme 3:**
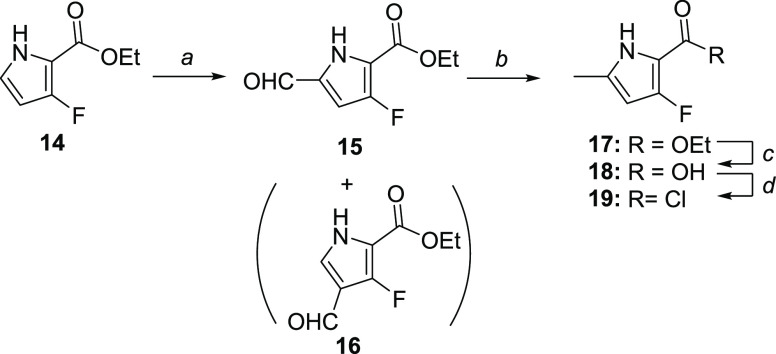
Synthesis of 3-Fluoro-5-methyl-1*H*-pyrrole-2-carbonyl Chloride **19** Reagents and conditions: (a) DMF, POCl_3_, 0 °C, 30 min, then **14**, 90 °C, overnight (25% yield); (b) Zn, 4 M HCl/dioxane, r.t., 40 min (20% yield); (c) 10 M NaOH(aq), EtOH, 90 °C, 5 h (82% yield); and (d) oxalyl chloride, dichloromethane, r.t., overnight (quant. yield).

Ethyl 3-fluoro-1*H*-pyrrole-2-carboxylate **14** has recently become commercially available at a reasonable price because it is a key building block for a drug candidate against hepatitis B virus.^22^ This was a good starting point for the synthesis of 3-fluoro-5-methyl-1*H*-pyrrole-2-carboxylic acid **18** (Scheme 3). The Vilsmeier–Haack formylation of **14** gave at 68% conversion a 43:57 mixture of 4- and 5-formylated regioisomers **16** and **15**, which were separated by flash chromatography. The regioisomers’ identity was assigned by ^19^F NMR as follows: 4-formyl isomer **16** features a singlet and 5-formyl isomer **15** features a doublet, ^3^*J*_F,H_ = 4 Hz, confirming the presence of a vicinal proton. Moreover, the ^13^C NMR peak of the formyl carbon of **15** is a singlet and that of **16** is a doublet, ^3^*J*_C,F_ = 2.8 Hz.

Based on the literature reports on the reduction of ester-containing formylpyrroles to methylpyrroles,^23^ we first attempted a BH_3_·THF-mediated reduction of **15**–**17**, which in this case yielded the intermediate alcohol; no full reduction was observed even after several days of stirring with periodical addition of excess BH_3_·THF. Other literature reports on aldehyde-to-methyl reduction in the presence of ester include a two-step Mozingo protocol via thioketal.^24^ Aiming to secure a convenient one-pot procedure, we opted for the modified Clemmensen reduction.^25^ A dioxane-soluble [ZnCl_2_(dioxane)_2_] complex^26^ was prepared by treating zinc dust with anhydrous 4 M HCl in dioxane. This proved to be a very efficient and reasonably selective reduction medium, delivering **17** after 40 min at r.t. in 20% isolated yield. Optimization of the reaction conditions and elucidation of the mechanism is beyond the aim of this study; however, we speculate that a dioxane-soluble Zn(II) species forms a zinc–ylide intermediate more efficiently compared to the classical heterogeneous Clemmensen reduction (Zn/Hg/HCl/H_2_O), allowing the reaction to proceed at room temperature.^27^ The side products are essentially a result of the zinc–ylide reaction with other present electrophiles (ester, aldehyde, and arylfluoride). Using the conditions developed for the synthesis of **13**, ester **17** was readily transformed to acyl chloride **19**.

**Scheme 4:**
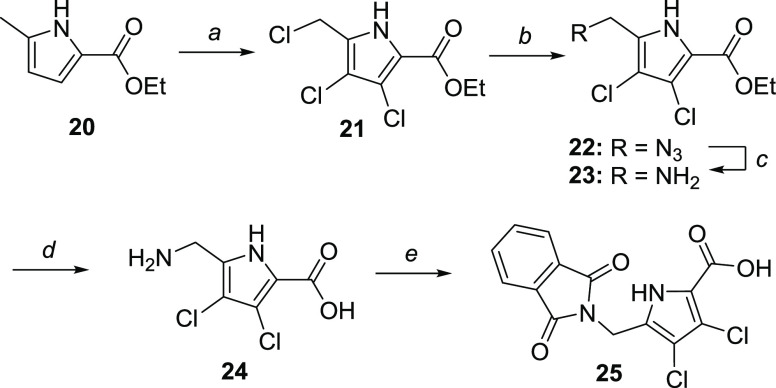
Synthesis of 3,4-Dichloro-5-phthalimidomethyl-1*H*-pyrrole-2-carboxylic Acid **25** Reagents and conditions: (a) SO_2_Cl_2_, CCl_4_, −5 °C, 5 h; (b) NaN_3_, KI (cat.), DMF, r.t., 1.5 h (95% yield); (c) PPh_3_, H_2_O/THF, r.t., 2.5 h (78% yield); (d) 10 M NaOH, EtOH, 90 °C, 4 h (quant. yield); and (e) phthalic anhydride, 130–180 °C, 1 h (55% yield).

Ethyl 5-chloromethyl-3,4-dichloro-1*H-*pyrrole-2-carboxylate **21** was prepared from commercially available **20** according to the literature procedure (Scheme 4).^18^ After conversion to azide **22** by KI-mediated nucleophilic substitution, the reduction of **22** to amine **23** was first attempted via Pd/C-catalyzed hydrogenation. This resulted in significant side-product formation, possibly via the nucleophilic attack of amine **23** to the electrophilic methylene moiety of **22**, and aryl dehalogenation, as apparent from the ^1^H NMR analysis of the crude reaction mixture. Avoiding the coexistence of amine and azide species during the reaction, we resorted to the milder Staudinger reduction,^28^ which furnished amine **23** in 78% isolated yield. Saponification to **24**, followed by phthalimide protection in neat phthalic anhydride gave **25** with 41% yield over four steps from **21**.

**Scheme 5:**
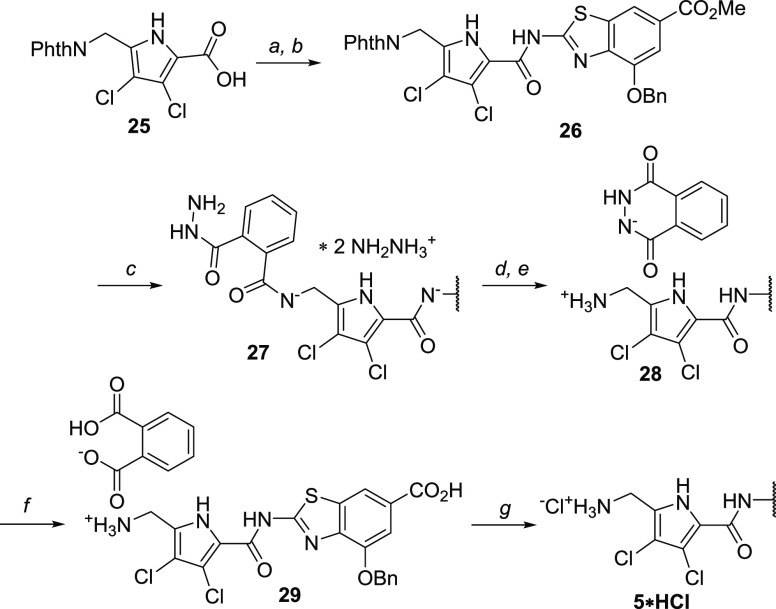
Synthesis of DNA Gyrase B Inhibitor **5·HCl** Reagents and conditions: (a) SOCl_2_, 75 °C, 1 h (quant. yield); (b) methyl 2-amino-4-benzyloxybenzo[*d*]thiazole-6-carboxylate, toluene, 130 °C, 24 h (81% yield); (c) NH_2_NH_2_·H_2_O, EtOH, 50 °C, 40 min; (d) HCl, MeOH, r.t., 15 min; (e) EtOH, 80 °C, 18 h (88% yield from **26**); (f) 4 M KOH, EtOH, 50 °C, 24 h then 1 M HCl to pH = 9; and (g) HCl, MeOH, r.t. (55% yield from **28**).

To showcase the usefulness of the prepared building blocks in medicinal chemistry, the synthesis of compound **5**, the analogue of antibacterial hit compound **1**, was tackled (Scheme 5). After the smooth coupling of the acyl chloride, prepared from **25** in neat thionyl chloride, with the 2-aminobenzothiazole building block^29^ in refluxing toluene, the deprotection step required some special attention. The formation of stable hydrazinium salt **27** was observed during the phthalimide deprotection, arguably due to the electron-withdrawing character of dichloropyrrole, which increases the acidity of the neighboring amides. It was crucial to first reprotonate the nitrogens of **27** to achieve complete deprotection after refluxing in ethanol overnight. Alkaline hydrolysis of phthalic hydrazide salt **28** yielded phthalate salt **29** and the anion was readily exchanged to the chloride salt of **5** by trituration with methanolic HCl.

Antibacterial hit compound **1** (*c* log *P* = 5.8) inhibited *Escherichia coli* DNA gyrase with IC_50_ < 10 nM, and compound **5** (*c* log *P* = 2.0) inhibited the same enzyme with IC_50_ < 10 nM. Moreover, **5** exhibits activity against *Staphylococcus aureus* (ATCC29213) with a minimal inhibitory concentration of 1 μg/mL. This confirms the hypothesis that the single-digit nanomolar inhibitory on-target activity coupled to the antibacterial activity can be retained while significantly reducing the lipophilicity by the modification of the pyrrole moiety.

**Scheme 6:**
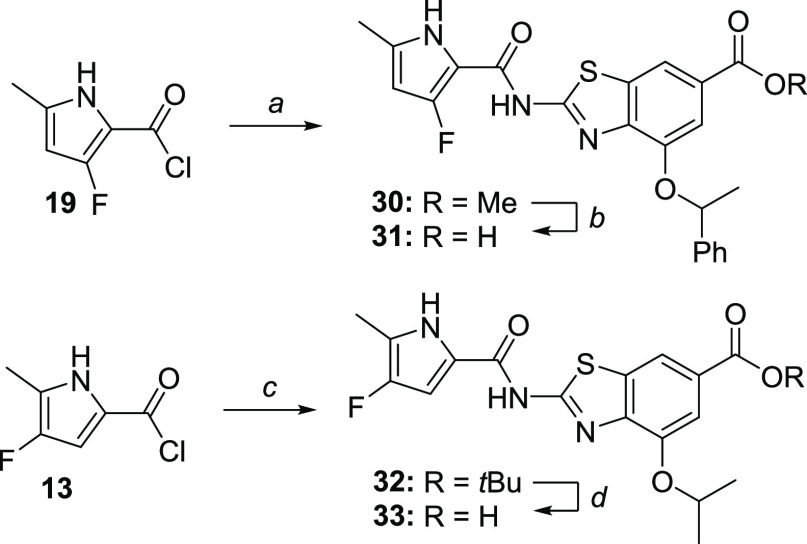
Synthesis of DNA Gyrase B Inhibitors **31** and **33** Reagents and conditions: (a) methyl 2-amino-4-(1-phenylethoxy)benzo[*d*]thiazole-6-carboxylate, toluene, 130 °C, 24 h (57% yield); (b) MeOH, 2 M NaOH, 40 °C, 48 h; (c) *tert*-butyl 2-amino-4-(2-propyloxy)benzo[*d*]thiazole-6-carboxylate, toluene, 130 °C, 24 h (79% yield); and (d) CF_3_COOH, DCM, r.t., 24 h (98% yield).

To explore the reactivity and bioactivity of the fluorinated pyrroles, two additional analogues of **1** were prepared (Scheme 6) and evaluated for their on-target and antibacterial activities. Thus, compounds **31** and **33** inhibited *E. coli* DNA gyrase with IC_50_ values of 32 and 150 nM, respectively, and possessed weak activity against *S. aureus* (ATCC29213) (**31**: MIC = 64 μg/mL; **33**: MIC > 64 μg/mL).

## 3 Conclusions

In summary, practical synthetic routes to four new halogen-doped pyrrole building blocks were developed, delivering the target compounds in sufficient quantities for further elaboration. Moreover, the transformation of the building blocks to potent DNA gyrase B inhibitors was demonstrated. Such building blocks are polar alternatives to molecular fragments found in naturally occurring or natural-product-inspired bioactive compounds and are useful in hit-to-lead optimization.

## 4 Experimental Section

### 4.1 General

Reactions were conducted under an inert atmosphere using anhydrous solvents when required. Analytical thin-layer chromatography (TLC) was performed on silica gel 60 F_254_ plates. Flash column chromatography was performed using silica gel 60 (40–63 μm). Melting points were determined on a Kofler apparatus and are uncorrected. ^1^H NMR (400 MHz, internal Me_4_Si), ^13^C NMR (101 MHz, internal CDCl_3_ or DMSO-*d*_6_), and ^19^F NMR (376 MHz, external CCl_3_F) spectra were recorded on a Bruker AVANCE III 400 spectrometer (Bruker Corporation, Billerica, MA) in a DMSO-*d*_6_ or CDCl_3_ solution. HRMS analysis was performed on a VG Analytical Autospec Q mass spectrometer (Fisons, VG Analytical, Manchester, U.K.).

### 4.2 Synthetic Procedures

#### 4.2.1 2-Trichloroacetyl-4-chloro-5-methyl-1*H*-pyrrole (**8**)

A mixture of 2-trichloroacetyl-5-methyl-1*H*-pyrrole (2.14 g, 9.44 mmol), *N*-chlorosuccinimide (1.26 g, 9.44 mmol), and dichloromethane (9.0 mL) was stirred at r.t. overnight. The reaction mixture was partitioned between EtOAc (50 mL) and water (50 mL), and the organic layer was washed with water and brine, dried (Na_2_SO_4_), and concentrated. The residue was recrystallized from dichloromethane to get the title compound as white crystals (1.51 g, 61% yield). Mp 140–142 °C (DCM). ^1^H NMR (400 MHz, CDCl_3_) δ 10.37 (s, 1H), 7.31 (d, *J* = 2.9 Hz, 1H), 2.41 (s, 2H). ^13^C NMR (101 MHz, CDCl_3_) δ 172.89, 137.01, 121.03, 119.78, 114.13, 94.87, 11.37. HRMS calcd for C_7_H_4_ONCl_4_ [M – H]^−^ 257.90525, found 257.90521.

#### 4.2.2 Ethyl 4-Fluoro-5-methyl-1*H*-pyrrole-2-carboxylate (**10**)

To a solution of ethyl 5-methyl-1*H*-pyrrole-2-carboxylate (2.00 g, 13.1 mmol) in acetonitrile (360 mL) and AcOH (72 mL) at 0 °C was added Selectfluor (9.25 g, 26.1 mmol), and the mixture was stirred at 0 °C for 2 h (full conversion by ^1^H NMR). The reaction mixture was partitioned between water (500 mL) and dichloromethane (500 mL), and the organic layer was washed with sat. NaHCO_3_ (aq) and brine, dried (Na_2_SO_4_), and concentrated to get the crude product. Crude products from two 2 g runs were combined and purified by flash chromatography, eluent hexane/EtOAc 4:1, to get the title compound (first eluting) as a colorless amorphous solid (288 mg, 6.5% yield). ^1^H NMR (400 MHz, CDCl_3_): δ 8.92 (bs, 1H), 6.54 (d, 1H, *J* = 4.0 Hz), 4.30 (q, 2H, *J* = 7.1 Hz), 2.24 (s, 3H), 1.34 (t, 3H, *J* = 7.1 Hz). ^13^C NMR (101 MHz, CDCl_3_) δ 161.3 (d, *J* = 3.3 Hz), 148.9 (d, *J* = 238.7 Hz), 117.6, 116.2 (d, *J* = 7.4 Hz), 102.3 (d, *J* = 15.6 Hz), 60.6, 14.6, 9.4 (d, *J* = 2.1 Hz). ^19^F NMR (376 MHz, CDCl_3_): δ −166.4. HRMS calcd for C_8_H_11_FNO_2_ [M + H]^+^ 172.0768, found 172.0769. This procedure was repeated several times, consistently yielding 4.5–6.5% of the title compound. A monocrystal suitable for single-crystal X-ray diffraction analysis was grown from dichloromethane/hexane.

#### 4.2.3 Ethyl 4-Acetoxy-5-methyl-1*H*-pyrrole-2-carboxylate (**11**)

Ethyl 4-acetoxy-5-methyl-1*H*-pyrrole-2-carboxylate was isolated as a second eluting product (see purification of **10** above), a white amorphous solid. ^1^H NMR (400 MHz, CDCl_3_): δ 10.10 (s, 1H), 6.72 (d, *J* = 2.8 Hz, 1H), 4.29 (q, *J* = 7.1 Hz, 1H), 2.24 (s, 1H), 2.16 (s, 1H), 1.32 (t, *J* = 7.1 Hz, 1H). ^13^C NMR (101 MHz, CDCl_3_): δ 169.23, 161.46, 134.49, 123.33, 117.80, 108.22, 60.46, 20.85, 14.55, 10.16. A monocrystal suitable for single-crystal X-ray diffraction analysis was grown from dichloromethane/hexane.

#### 4.2.4 4-Fluoro-5-methyl-1*H*-pyrrole-2-carboxylic Acid (**12**)

A solution of the above ester **10** (280 mg, 1.07 mmol) in abs. EtOH (15 mL) and 10 M NaOH (3.0 mL) was stirred at 90 °C under an argon atmosphere for 3 h, and then the reaction mixture was concentrated under reduced pressure. The residue was acidified to pH = 3 by adding 4 M HCl, and the precipitate was collected, washed with water, and air-dried to get the title compound as a light brown amorphous solid (177 mg, 76% yield). ^1^H NMR (400 MHz, DMSO-*d*_6_) δ 12.25 (s, 1H), 11.51 (s, 1H), 6.45 (dd, *J* = 2.9, 1.1 Hz, 1H), 2.13 (s, 3H). ^19^F NMR (376 MHz, DMSO-*d*_6_) δ −167.66 to −167.70 (m). ^13^C NMR (101 MHz, DMSO-*d*_6_) δ 161.51 (d, *J* = 3.3 Hz), 147.77 (d, *J* = 235.0 Hz), 116.90 (d, *J* = 25.1 Hz), 116.15 (d, *J* = 7.3 Hz), 100.94 (d, *J* = 14.9 Hz), 8.70 (d, *J* = 2.2 Hz). HRMS calcd for C_6_H_5_O_2_NF [M – H]^−^142.03098, found 142.03001.

#### 4.2.5 4-Fluoro-5-methyl-1*H*-pyrrole-2-carbonyl Chloride (**13**)

A suspension of the above acid (75 mg, 0.52 mmol) in dry dichloromethane (5.2 mL) and oxalyl chloride (0.45 mL, 5.2 mmol) was stirred at r.t. overnight. The resulting clear solution was concentrated under reduced pressure to get the title compound as a light brown amorphous solid (quant. yield). ^1^H NMR (400 MHz, CDCl_3_) δ 8.66 (s, 1H), 6.83 (d, *J* = 2.8 Hz, 1H), 2.30 (s, 3H). ^19^F NMR shows no clearly identifiable peak.

#### 4.2.6 Ethyl 3-Fluoro-5-formyl-1*H*-pyrrole-2-carboxylate (**15**) and Ethyl 3-Fluoro-4-formyl-1*H*-pyrrole-2-carboxylate (**16**)

To DMF (8.9 mL, 115 mmol) at 0 °C under Ar was added POCl_3_ (1.95 mL, 21.0 mmol). After stirring at 0 °C for 30 min, a solution of ethyl 3-fluoro-1*H*-pyrrole-2-carboxylate **14** (3.00 g, 19.1 mmol) in DMF (29 mL) was added, and the resulting solution was stirred at 90 °C overnight. The reaction mixture was cooled to r.t. and poured onto ice (100 mL), the pH was adjusted to 9 by adding 2 M NaOH (aq) and the product was extracted to Et_2_O (3 × 200 mL). The combined organic layers were washed with brine, dried (Na_2_SO_4_), and concentrated to get the crude product, containing **14**, 5-formylated (**15**), and 4-formylated product (**16)** in a 42:34:24 ratio (by ^1^H NMR). The three compounds were separated by column chromatography, eluent dichloromethane, then hexane/EtOAc 2:1 to yield **14** (983 mg, a white amorphous solid), **15** (869 mg, 25% yield, an orange amorphous solid), and **16** (724 mg, a yellow amorphous solid).

Compound **15**: ^1^H NMR (400 MHz, CDCl_3_) δ 9.88 (s, 1H), 9.42 (br s, 1H), 7.36 (app t, *J* = 3.9 Hz, 1H), 4.39 (q, *J* = 7.1 Hz, 2H), 1.39 (t, *J* = 7.1 Hz, 3H). ^19^F NMR (376 MHz, CDCl_3_) δ −150.04 (d, *J* = 4 Hz). ^13^C NMR (101 MHz, CDCl_3_) δ 183.57, 160.22 (d, *J* = 3.5 Hz), 152.93 (d, *J* = 269.6 Hz), 124.59 (d, *J* = 3.6 Hz), 115.00 (d, *J* = 8.5 Hz), 109.40 (d, *J* = 18.1 Hz), 61.48, 14.35. HRMS calcd for C_8_H_9_O_3_NF [M + H]^+^ 186.05610, found 186.05611.

Compound **16**: ^1^H NMR (400 MHz, CDCl_3_) δ 9.59 (s, 1H), 9.47 (s, 1H), 6.71–6.54 (m, 1H), 4.41 (q, *J* = 7.1 Hz, 2H), 1.40 (t, *J* = 7.1 Hz, 3H). ^19^F NMR (376 MHz, CDCl_3_) δ −146.95. ^13^C NMR (101 MHz, CDCl_3_) δ 180.38 (d, *J* = 2.8 Hz), 159.67 (d, *J* = 3.9 Hz), 153.06 (d, *J* = 260.3 Hz), 129.65 (d, *J* = 4.4 Hz), 113.90 (d, *J* = 20.0 Hz), 105.40 (d, *J* = 14.2 Hz), 61.75, 14.32.

#### 4.2.7 Ethyl 3-Fluoro-5-methyl-1*H*-pyrrole-2-carboxylate (**17**)

To a solution of the above aldehyde **15** (500 mg, 2.70 mmol) in 4 M HCl in dioxane (27 mL) was added Zn dust (1.78 g, 27.0 mmol) portionwise at room temperature over 2 min. The reaction is slightly exothermic and performs better if no external cooling is applied. After 40 min (full conversion by TLC), the reaction mixture was concentrated under reduced pressure and filtered through a plug of silica, eluting with EtOAc (200 mL), and concentrated to 5 mL, and the colorless crystals were filtered off and washed with EtOAc (^1^H NMR analysis revealed only a dioxane peak; it is likely a dioxane-soluble [ZnCl*_x_*(dioxane)*_x_*] complex involved in the aldehyde reduction). The filtrate was diluted with EtOAc (200 mL), washed with water (2 × 100 mL), NaHCO_3_ (100 mL), and brine, dried (Na_2_SO_4_), and concentrated to get the crude product (486 mg), containing 30 mol % of the desired product (by ^19^F NMR). The pure **17** was isolated by flash chromatography on silica, eluent hexane/EtOAc 4:1 (first eluting compound) as a colorless oil containing residual EtOAc (by ^1^H NMR) (94 mg, 20% yield) and was used as such in the next step. ^1^H NMR (400 MHz, CDCl_3_) δ 8.32 (br s, 1H), 6.58 – 6.51 (m, 1H), 4.33 (q, *J* = 7.1 Hz, 2H), 2.02 (s, 3H), 1.36 (t, *J* = 7.1 Hz, 3H). ^19^F NMR (376 MHz, CDCl_3_) δ −154.53 (d, *J* = 4.7 Hz). HRMS calcd for C_8_H_11_O_2_NF [M + H]^+^ 172.07683, found 172.07625.

#### 4.2.8 3-Fluoro-5-methyl-1*H*-pyrrole-2-carboxylic Acid (**18**)

A solution of the above ester **17** (80 mg, 0.47 mmol) in abs. EtOH (3.5 mL) and 10 M NaOH (0.63 mL) was heated at 90 °C under Ar for 2 h, and then it was concentrated under reduced pressure. The oily residue was dissolved in water (2 mL), and 4 M HCl (1.6 mL) was added. The obtained precipitate was collected, washed with water, and air-dried to get the title compound as a white amorphous powder (55 mg, 82% yield). ^1^H NMR (400 MHz, DMSO-*d*_6_) δ 12.32 (s, 1H), 11.25 (s, 1H), 6.66 (app t, *J* = 4.4 Hz, 1H), 1.93 (s, 3H). ^19^F NMR (376 MHz, DMSO-*d*_6_) δ −156.45. ^13^C NMR (101 MHz, DMSO-*d*_6_) δ 160.49 (d, *J* = 3.5 Hz), 152.39 (d, *J* = 252.4 Hz), 119.46 (d, *J* = 6.4 Hz), 106.77 (d, *J* = 18.3 Hz), 105.46 (d, *J* = 13.9 Hz), 7.26. HRMS calcd. for C_6_H_7_O_2_NF [M + H]^+^ 144.04553, found 144.04552.

#### 4.2.9 3-Fluoro-5-methyl-1*H*-pyrrole-2-carbonyl Chloride (**19**)

A suspension of the above acid **18** (50 mg, 0.35 mmol) in dry dichloromethane (3.5 mL) and oxalyl chloride (0.3 mL, 3.5 mmol) was stirred at r.t. overnight. The resulting clear solution was concentrated under reduced pressure to get the title compound as a light brown amorphous solid (quant. yield). ^1^H NMR (400 MHz, CDCl_3_) δ 8.51 (br s, 1H), 6.81–6.76 (m, 1H), 2.04 (d, *J* = 0.9 Hz, 3H). ^19^F NMR showed no clearly identifiable peak.

#### 4.2.10 Ethyl 5-(azidomethyl)-3,4-dichloro-1*H*-pyrrole-2-carboxylate (**22**)

To a solution of ethyl 5-(chloromethyl)-3,4-dichloro-1*H*-pyrrole-2-carboxylate **21** (2.00 g, 7.80 mmol) in DMF (16 mL) was added NaN_3_ (1.00 g, 15.6 mmol), followed by KI (130 mg, 0.78 mmol). The resulting suspension was vigorously stirred at r.t. for 1.5 h, and then it was poured into water (100 mL). The white precipitate was collected, washed with water, and air-dried to yield the title compound as a white amorphous powder (1.95 g, 95% yield). ^1^H NMR (400 MHz, CDCl_3_) δ 9.57 (s, 1H), 4.45 (s, 2H), 4.40 (q, *J* = 7.1 Hz, 2H), 1.41 (t, *J* = 7.1 Hz, 3H). ^13^C NMR (101 MHz, CDCl_3_) δ 160.08, 126.48, 118.30, 117.72, 113.26, 61.66, 45.22, 14.45. HRMS calcd for C_8_H_7_O_2_N_4_Cl_2_ [M – H]^−^ 260.99515, found 260.99529.

#### 4.2.11 Ethyl 5-(aminomethyl)-3,4-dichloro-1*H*-pyrrole-2-carboxylate (**23**)

To a solution of the above azide **22** (1.70 g, 6.46 mmol) in THF/H_2_O 10:1 (35 mL) was added PPh_3_ (3.39 g, 12.9 mmol) at r.t. The resulting amber solution was stirred at r.t. (caution: gas evolution) for 2.5 h, and then it was concentrated under reduced pressure. The oily residue was partitioned between EtOAc (200 mL) and 0.5 M HCl (300 mL). The water layer was brought to pH = 11 by adding 2 M NaOH. The precipitate was collected, washed with water, and dried in vacuo to yield the title compound as a white amorphous powder (1.20 g, 78%). ^1^H NMR (400 MHz, CDCl_3_) δ 9.87 (s, 1H), 4.36 (q, *J* = 7.1 Hz, 2H), 3.99 (s, 2H), 1.63 (s, 2H), 1.38 (t, *J* = 7.1 Hz, 3H). ^13^C NMR (101 MHz, CDCl_3_) δ 160.16, 133.16, 117.50, 116.76, 110.24, 61.02, 36.85, 14.49. HRMS calcd for C_8_H_9_O_2_N_2_Cl_2_ [M – H]^−^ 235.00466, found 235.00459.

#### 4.2.12 5-(Aminomethyl)-3,4-dichloro-1*H*-pyrrole-2-carboxylic Acid (**24**)

A mixture of the above ester **23** (1.42 g, 6.00 mmol), abs. EtOH (60 mL), and 10 M NaOH (10.8 mL) was stirred at 90 °C under argon for 3 h. The reaction mixture was concentrated under reduced pressure, dissolved in water (20 mL), filtered through cotton, and cooled to 0 °C. Conc. HCl(aq) (7.5 mL) was added, followed by 2 M HCl to adjust the pH to 8. The precipitate was collected, washed with water, and air-dried to yield the title compound as a white amorphous powder (1.3 g, quant. yield). ^1^H NMR (400 MHz, DMSO-*d*_6_) δ 3.91 (s, 2H). HRMS calcd for C_6_H_5_O_2_N_2_Cl_2_ [M – H]^−^ 206.97336, found 206.97342.

#### 4.2.13 5-(Phthalimidomethyl)-3,4-dichloro-1*H*-pyrrole-2-carboxylic Acid (**25**)

A homogeneous mixture of the above amino acid **24** (300 mg, 1.44 mmol) and powdered phthalic anhydride (213 mg, 1.44 mmol) in a 25 mL round-bottom flask was heated on an oil bath under a stream of argon from 150 to 180 °C for 15 min while stirring with a magnetic stirrer at 100 rpm and agitating the flask manually. The temperature was kept at 180 °C for 30 min during which the reaction mixture caked. After cooling to r.t., the crude product was triturated successively with dichloromethane, EtOAc, and 1 M HCl, washed with water, and air-dried to get the title compound as a light gray amorphous solid (270 mg, 55% yield). ^1^H NMR (400 MHz, DMSO-*d*_6_) δ 13.04 (s, 1H), 12.57 (s, 1H), 8.01–7.76 (m, 4H), 4.79 (s, 2H). ^13^C NMR (101 MHz, DMSO-*d*_6_) δ 167.31, 160.04, 134.42, 131.83, 127.18, 123.14, 117.38, 114.98, 109.12, 33.00. HRMS calcd for C_14_H_7_O_4_N_2_Cl_2_ [M – H]^−^ 336.97884, found 336.97929.

#### 4.2.14 Methyl 4-(benzyloxy)-2-(3,4-dichloro-5-((1,3-dioxoisoindolin -2-yl)methyl)-1*H*-pyrrole-2-carboxamido)benzo[*d*]thiazole-6-carboxylate (**26**)

A suspension of the above carboxylic acid **25** (110 mg, 0.324 mmol) in SOCl_2_ (1 mL) was refluxed for 1 h and then concentrated under reduced pressure. To the solid residue were added methyl 2-amino-4-(benzyloxy)benzo[*d*]thiazole-6-carboxylate (102 mg, 0.324 mg) and normal grade toluene (6.5 mL), and the resulting suspension was refluxed overnight. After cooling to r.t., the precipitate was collected, washed with toluene, and air-dried to get the title compound as a gray amorphous solid (165 mg, 81% yield). ^1^H NMR (400 MHz, DMSO-*d*_6_) δ 12.44 (s, 1H), 12.39 (s, 1H), 8.30 (s, 1H), 7.95–7.85 (m, 4H), 7.61 (s, 1H), 7.55–7.50 (m, 2H), 7.46–7.34 (m, 3H), 5.30 (s, 2H), 4.82 (s, 2H), 3.88 (s, 3H). HRMS calcd for C_30_H_19_O_6_N_4_Cl_2_S [M – H]^−^ 633.04078, found 633.04098.

#### 4.2.15 (5-((4-(Benzyloxy)-6-(methoxycarbonyl)benzo[*d*]thiazol-2-yl)carbamoyl)-3,4-dichloro-1*H*-pyrrol-2-yl)methanaminium 1,4-Dioxo-3,4-dihydro-1*H*-phthalazin-2-ide (**28**)

To a suspension of **26** (100 mg, 0.157 mmol) in abs. EtOH (3.2 mL) was added 80% aqueous hydrazine hydrate (0.1 mL, 10 equiv). After stirring at 50 °C for 30 min, full conversion to **27** was achieved; ^1^H NMR (400 MHz, DMSO-*d*_6_) δ 8.66 (t, *J* = 5.6 Hz, 1H), 7.95 (d, *J* = 1.6 Hz, 1H), 7.56–7.52 (m, 2H), 7.51–7.45 (m, 4H), 7.45–7.39 (m, 3H), 7.37–7.31 (m, 1H), 5.29 (s, 2H), 4.43 (d, *J* = 5.6 Hz, 2H), 3.83 (s, 3H). The reaction mixture was concentrated under reduced pressure, suspended in MeOH (5 mL), treated with 37% HCl(aq) (three drops), and concentrated to get a white residue. ^1^H NMR (400 MHz, DMSO-*d*_6_) δ 12.50–12.36 (m, 2H), 11.41 (s, 1H), 10.63 (s, 2H), 9.10 (t, *J* = 5.4 Hz, 1H), 8.30 (d, *J* = 1.4 Hz, 1H), 7.76–7.71 (m, 1H), 7.67–7.58 (m, 3H), 7.57–7.48 (m, 3H), 7.47–7.41 (m, 2H), 7.40–7.35 (m, 1H), 5.33 (s, 2H), 4.46 (d, *J* = 5.3 Hz, 2H), 3.89 (s, 3H). This reprotonated compound was suspended in 96% EtOH and stirred at 80 °C overnight, and then the reaction mixture was concentrated and triturated with acetone to get the title compound **28** as a gray amorphous solid (90 mg, 88% yield). ^1^H NMR (400 MHz, DMSO-*d*_6_) δ 12.66 (s, 1H), 12.51 (s, 1H), 11.64 (s, 1H), 8.42–8.28 (m, 4H), 8.19–7.98 (m, 2H), 7.89 (dd, *J* = 5.9, 3.3 Hz, 2H), 7.64 (d, *J* = 1.5 Hz, 1H), 7.58–7.49 (m, 2H), 7.48–7.41 (m, 2H), 7.41–7.35 (m, 1H), 5.34 (s, 2H), 4.06 (q, *J* = 5.2 Hz, 2H), 3.89 (s, 3H). HRMS calcd for C_22_H_19_O_4_N_4_Cl_2_S [M]^+^ 505.04986, found 505.04879; calcd for C_8_H_5_O_2_N_2_ 161.03565 [M]^−^, found 161.03471.

#### 4.2.16 (5-((4-(Benzyloxy)-6-carboxybenzo[*d*]thiazol-2-yl)carbamo-yl)-3,4-dichloro-1*H*-pyrrol-2-yl)methanaminium 2-Carboxybenzoate (**29**)

A mixture of the above ester **28** (50 mg, 0.075 mmol), abs. EtOH (1.0 mL), and 4 M KOH (0.15 mL) was stirred at 80 °C for 2 h and then at 50 °C overnight. The reaction mixture was concentrated, water (1 mL) was added, and the solids were filtered off. The filtrate was brought to pH = 6 by adding 1 M HCl and cooled to 0 °C. The amorphous precipitate was collected, washed with water, and air-dried. ^1^H NMR (400 MHz, DMSO-*d*_6_) δ 8.18 (s, 1H), 8.08 (s, 2H), 7.96–7.82 (m, 3H), 7.60–7.50 (m, 3H), 7.48–7.31 (m, 3H), 5.30 (s, 2H), 3.94 (s, 2H), exchangeable protons were not observed.

#### 4.2.17 (5-((4-(Benzyloxy)-6-carboxybenzo[*d*]thiazol-2-yl)carbamo-yl)-3,4-dichloro-1*H*-pyrrol-2-yl)methanaminium Chloride (**5·HCl**)

The above phthalate salt **29** was triturated with a fresh solution of HCl in MeOH (2 × 1 mL) (prepared by adding two drops of 37% HCl (aq) to absolute methanol (5 mL) (methanol was chosen as a solvent because of the known good solubility of phthalic acid in methanol)) to get the title compound as a brown amorphous powder (22 mg, 55% yield). ^1^H NMR (400 MHz, DMSO-*d*_6_) δ 13.05 (s, 1H), 12.64 (s, 1H), 12.47 (s, 1H), 8.39–8.20 (m, 4H), 7.64 (d, *J* = 1.3 Hz, 1H), 7.55 (d, *J* = 7.2 Hz, 2H), 7.47–7.42 (m, 2H), 7.41–7.36 (m, 1H), 5.33 (s, 2H), 4.07 (q, *J* = 5 Hz, 2H). ^13^C NMR (101 MHz, DMSO-*d*_6_) representative peaks: δ 167.46, 137.04, 128.91, 128.76, 128.58, 127.47, 125.72, 116.82, 115.03, 112.68, 109.63, 70.58, 33.42. HRMS calcd for C_21_H_17_O_4_N_4_Cl_2_S [M – Cl]^+^ 491.0348, found 491.0334.

#### 4.2.18 Methyl 2-(3-fluoro-5-methyl-1*H*-pyrrole-2-carboxamido)-4-(1-phenylethoxy)benzo[*d*]thiazole-6-carboxylate (**30**)

A suspension of **19** (56 mg, 0.35 mmol) and methyl 2-amino-4-(1-phenylethoxy)benzo[*d*]thiazole-6-carboxylate (114 mg, 0.349 mmol) in toluene (7 mL) was refluxed overnight, equipped with a CaCl_2_ tube. After cooling to r.t., the precipitate was collected and washed with toluene to get the title compound as a light gray amorphous powder (91 mg, 57% yield). ^1^H NMR (400 MHz, DMSO-*d*_6_) δ 12.23 (s, 1H), 11.37 (s, 1H), 8.17 (d, *J* = 1.5 Hz, 1H), 7.49–7.42 (m, 2H), 7.40–7.31 (m, 3H), 7.29–7.25 (m, 1H), 6.91 (app t, *J* = 4.2 Hz, 1H), 5.80 (q, *J* = 6.1 Hz, 1H), 3.80 (s, 3H), 1.99 (s, 3H), 1.64 (d, *J* = 6.3 Hz, 3H). ^19^F NMR (376 MHz, DMSO-*d*_6_) δ −153.97 (d, *J* = 4 Hz). HRMS calcd for C_23_H_19_O_4_N_3_FS 452.10858; found 452.10924 [M – H]^−^.

#### 4.2.19 2-(3-Fluoro-5-methyl-1*H*-pyrrole-2-carboxamido)-4-(1-phenylethoxy)benzo[*d*]thiazole-6-carboxylic Acid (**31**)

A solution of the above ester **30** (70 mg, 0.154 mmol) in MeOH (3.0 mL) and 2 M NaOH (0.40 mL) was stirred at 40 °C overnight. NaOH (2 M, 0.40 mL) was added and stirred another night, and then the reaction mixture was concentrated. The residue was suspended in water (2 mL), the pH was adjusted to 2 by adding 4 M HCl, and the precipitate was collected, washed with water, air-dried, and triturated with MeOH to get the title compound as a beige amorphous solid (55 mg, 81% yield). ^1^H NMR (400 MHz, DMSO-*d*_6_) δ 12.82 (s, 1H), 12.20 (s, 1H), 11.37 (s, 1H), 8.13 (s, 1H), 7.52–7.40 (m, 2H), 7.40–7.31 (m, 3H), 7.31–7.20 (m, 1H), 6.91 (app t, *J* = 4.2 Hz, 1H), 5.79 (q, *J* = 6.3 Hz, 1H), 1.99 (s, 3H), 1.64 (d, *J* = 6.3 Hz, 3H). ^19^F NMR (376 MHz, DMSO-*d*_6_) δ −154.06 (d, *J* = 4 Hz). ^13^C NMR (101 MHz, DMSO-*d*_6_) δ 166.92, 159.75, 156.78, 154.36, 151.82, 148.98, 142.57, 132.83, 128.61, 127.55, 126.31, 125.53, 120.98 (d, *J* = 5.4 Hz), 115.90, 110.44, 107.83 (d, *J* = 15.0 Hz), 106.18 (d, *J* = 13.7 Hz), 75.48, 24.33, 7.17. HRMS calcd for C_22_H_17_O_4_N_3_FS 438.09293; found 438.09338 [M – H]^−^.

#### 4.2.20 *tert*-Butyl 2-(4-fluoro-5-methyl-1*H*-pyrrole-2-carboxamido)-4-isopropoxybenzo[*d*]thiazole-6-carboxylate (**32**)

A suspension of **13** (58 mg, 0.36 mmol) and methyl 2-amino-4-(2-propyloxy)benzo[*d*]thiazole-6-carboxylate (93 mg, 0.30 mmol) in toluene (7 mL) was stirred at 130 °C overnight. The gray amorphous precipitate was collected and washed with toluene. Yield: 79% (104 mg). ^1^H NMR (400 MHz, DMSO-*d*_6_) δ 12.66 (s, 1H), 11.85 (s, 1H), 8.13 (d, *J* = 1 Hz, 1H), 7.44 (d, *J* = 1 Hz, 1H), 7.26 (d, *J* = 2.3 Hz, 1H), 4.95–4.84 (m, 1H), 2.20 (s, 3H), 1.36 (d, *J* = 6.0 Hz, 6H). MS 432.2 [M – H]^−^.

#### 4.2.21 2-(4-Fluoro-5-methyl-1*H*-pyrrole-2-carboxamido)-4-isopropoxybenzo[*d*]thiazole-6-carboxylic Acid (**33**)

The above *tert*-butyl ester **32** (75 mg, 0.173 mmol) was suspended in dichloromethane (3 mL). Trifluoroacetic acid (0.13 mL, 1.73 mmol) was added and the suspension turned into a brown solution. The reaction mixture was stirred overnight at room temperature. The solvent was removed under reduced pressure and the residue was triturated with methanol to give **33** as an amorphous solid. Yield: 98% (64 mg). ^1^H NMR (400 MHz, DMSO-*d*_6_) δ 12.92 (s, 1H), 12.65 (s, 1H), 11.84 (s, 1H), 8.17 (d, *J* = 1.2 Hz, 1H), 7.48 (s, 1H), 7.26 (d, *J* = 2.7 Hz, 1H), 4.89 (hept, *J* = 6 Hz, 1H), 2.19 (s, 3H), 1.36 (d, *J* = 6 Hz, 6H). HRMS calcd for C_17_H_17_O_4_N_3_FS [M + H]^+^ 378.09183, found 378.09203.

### 4.3 Biological Assays

#### 4.3.1 Determination of Inhibitory Activity on *E. coli*. DNA Gyrase

The supercoiling assay for the determination of IC_50_ values was performed according to previously reported procedures.^30^

## Supporting Information Available

Additional experimental data; copies of ^1^H and ^13^C and 2D NMR spectra; and single-crystal X-ray diffraction analysis (PDF)
Compound **10** (CIF)
Compound **11** (CIF)
checkCIF/PLATON report (PDF)

## Supplementary Material

ao1c00331_si_001.pdf

ao1c00331_si_002.cif

ao1c00331_si_003.cif

## Accession Codes

CCDC 2022198 and 2022199 contain the supplementary crystallographic data for this paper. These data can be obtained free of charge via www.ccdc.cam.ac.uk/data_request/cif, or by emailing data_request@ccdc.cam.ac.uk, or by contacting The Cambridge Crystallographic Data Centre, 12 Union Road, Cambridge CB21EZ, UK; fax: +44 1223 336033.
