# Supplementary material for: Practical Synthesis and Application of Halogen-Doped Pyrrole Building Blocks
Source: ACS Omega. 2021 Mar 30;6(14):9723–30. doi: 10.1021/acsomega.1c00331 (PMC8047689; doi:10.1021/acsomega.1c00331)
Supplement: Supplementary file 1 — ao1c00331_si_001.pdf [file ao1c00331_si_001.pdf]

Supporting Information

Practical synthesis and application of halogen-doped pyrrole building blocks

Andrej Emanuel Cotman,[a] Thomas Guérin,[b] Ivana Kovačević,[c] Davide Benedetto Tiz,[a] Martina Durcik,[a] Federica Fulgheri,[a] Štefan Možina,[a] Daniela Secci,[a] Maša Sterle,[a] Janez Ilaš,[a] Anamarija Zega,[a] Nace Zidar,[a] Lucija Peterlin Mašič,[a] Tihomir Tomašič,[a] Frédéric R. Leroux,[b] Gilles Hanquet,[b] Danijel Kikelj*[a]

[a] University of Ljubljana, Faculty of Pharmacy, Aškerčeva cesta 7, 1000 Ljubljana, Slovenia
[b] Université de Strasbourg, CNRS, UMR 7042-LIMA, ECPM, 25 Rue Becquerel, Strasbourg 67087, France
[c] University of Novi Sad, Faculty of Sciences, Department of Chemistry, Biochemistry and Environmental Protection, Trg Dositeja Obradovića 3 , 21000 Novi Sad, Serbia

Table of contents

Screening of the electrophilic fluorination reaction conditions S2

^{1}H, ^{13}C and 2D NMR spectra of new compounds S3

Single crystal X-ray diffraction analysis S21

References... S25

Screening of the electrophilic fluorination reaction conditions

Table S1. Electrophilic fluorination of ethyl 5-methyl-1*H*-pyrrole-2-carboxylate **9**.[a]

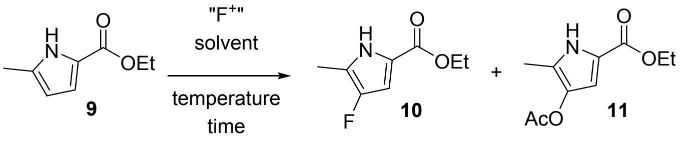

Entry	Reaction conditions	Products (Yield, %)[b]
1	NFSI (1 eq), $AlCl_3$ (0.3 eq), DCM (0.13 M), 150 h, rt	**10** in CM
2	NFSI (1.1 eq), $AlCl_3$ (0.3 eq), DCM (0.13) M, 45 min, 40 °C	**10** in CM
3	NFSI (1.1 eq), $AlCl_3$ (0.3 eq), DCM, (0.13 M), 65 h, 40 °C	**10** in CM
4	NFSI (4 eq), $AlCl_3$ (0.5 eq), DCM, (0.13 M), 20 h, 40 °C	**10** in CM
5	SelectFluor® (1.5 eq), ACN (0.1M), 35 min, 0 °C	**10** (2%)
6	SelectFluor® (1.5 eq), ACN (0.1M), 3 h, rt, 1 h, 50 °C	**10** (4%)
7	SelectFluor® (1.5 eq), ACN (0.1M), 48 h, 80 °C	**10** (3%)
8	SelectFluor® (4.5 eq), ACN (0.02 M), $Pd(OAc)_2$ (0.1 eq)	**10** (4%)
9	SelectFluor® (1.5 eq), ACN/AcOH (5:1, 0.03 M), 15 h, 70 °C	**10** (8%), **11** (7%)
10	SelectFluor® (1.5 eq), ACN/AcOH (10:1, 0.04 M), 18 h, rt	**10** (6%), **11** (8%)
11	SelectFluor® (1.5 eq), ACN/AcOH (5:1, 0.03 M), 13 h, 0 °C	**10** (10%), **11** (17%)
12	SelectFluor® (1.5 eq), ACN/AcOH (5:1, 0.03 M), 35 min, 0 °C	**10** (22%), **11** (22%)
13	SelectFluor® (1.5 eq), ACN/TFA (5:1, 0.03 M), 35 min, 0 °C	**10** (3.6%), **11** (n.a.)

a) The reactions were conducted in dry solvents under argon atmosphere on 0.5 mmol scale. *b)* Isolated yield. NFSI = *N*-Fluorodibenzenesulfonimide; DCM = dichloromethane; CM = complex mixture; ACN = acetonitrile; TFA = trifluoroacetic acid; n.a. = not applicable.

^{1}H, ^{13}C and 2D NMR spectra of new compounds

5-Methyl-4-chloro-2-trichloroacetyl-1*H*-pyrrole (**8**), ^{1}H NMR (400 MHz, $CDCl_3$):

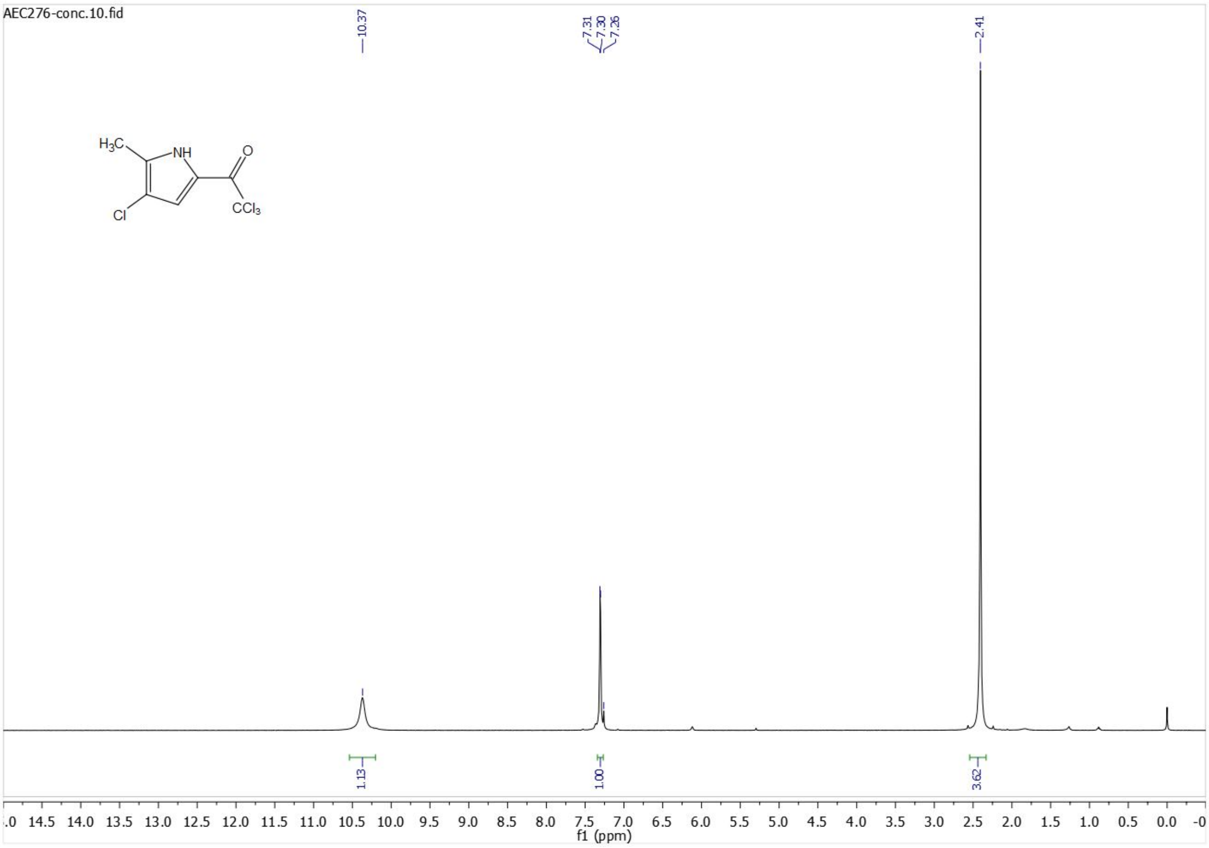

5-Methyl-4-chloro-2-trichloroacetyl-1*H*-pyrrole (**8**), ^{13}C NMR (100 MHz, $CDCl_3$):

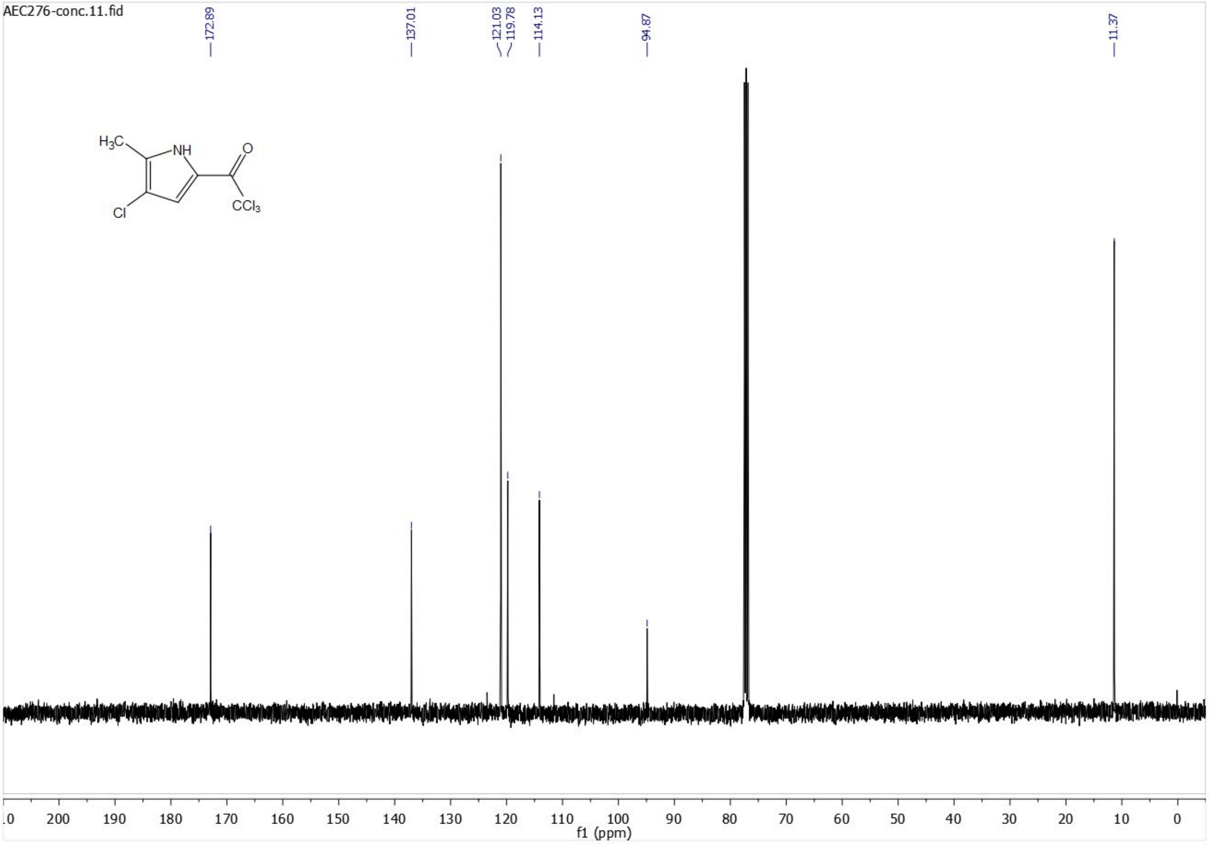

5-Methyl-4-chloro-2-trichloroacetyl-1*H*-pyrrole (**8**), ^{1}H NMR (400 MHz, DMSO-d_6):

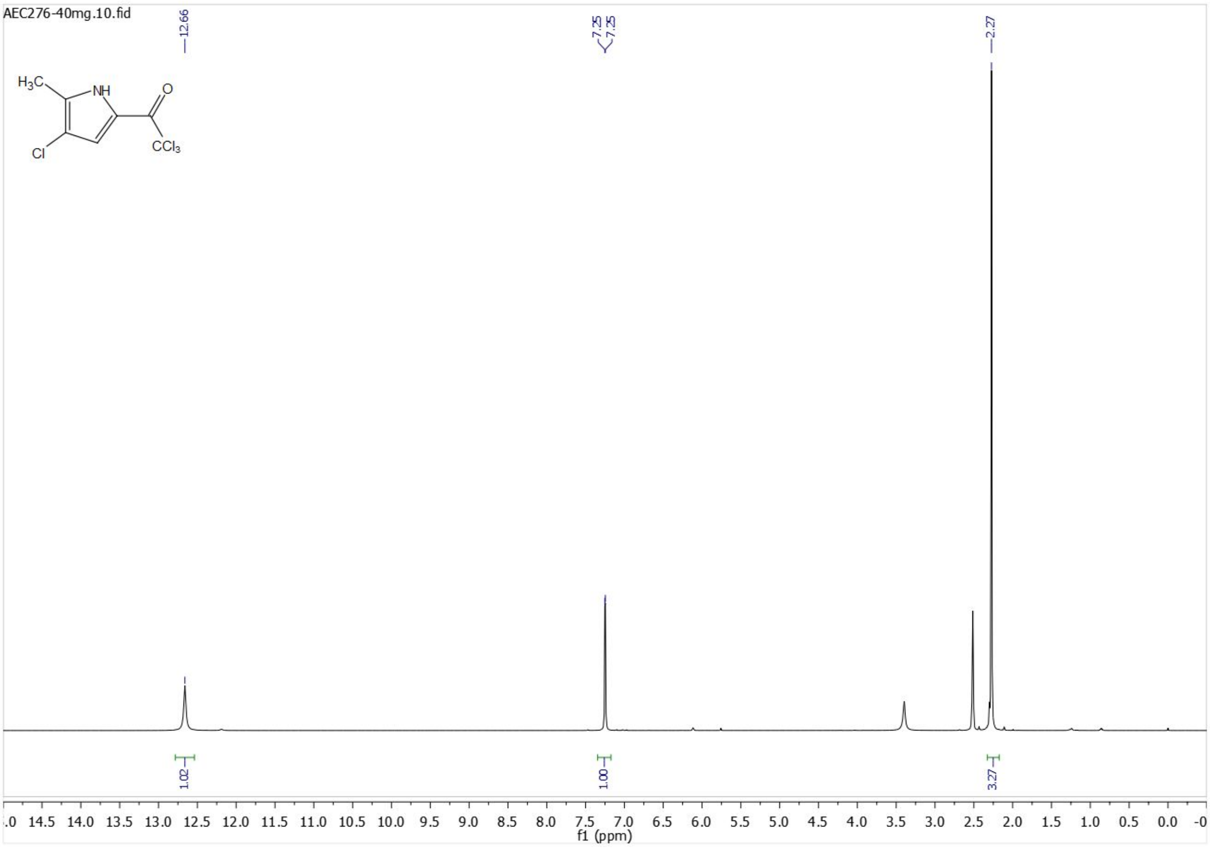

5-Methyl-4-chloro-2-trichloroacetyl-1*H*-pyrrole (**8**), ^{13}C NMR (100 MHz, DMSO-d_6):

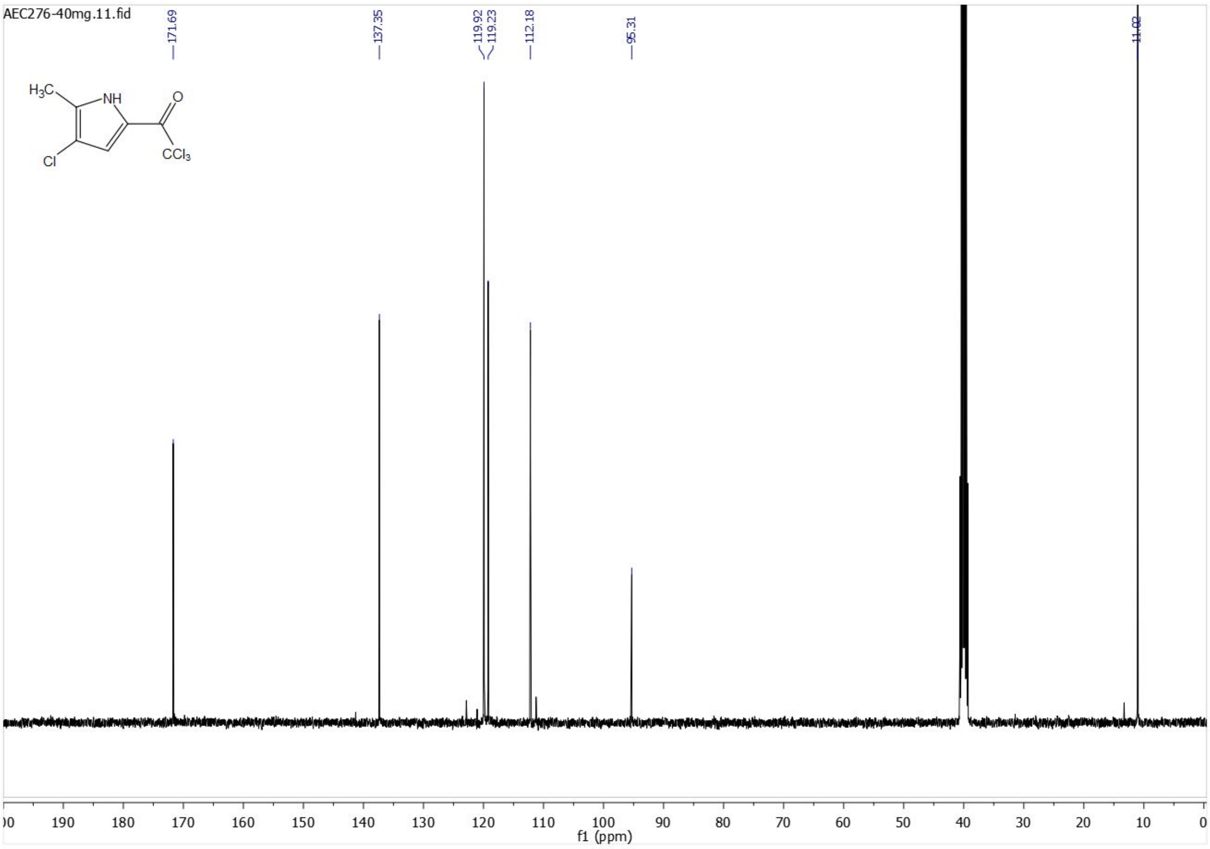

2D NMR analysis of **8**, observations: The aromatic CH (C3) has a ^{13}C NMR peak at 120.1 ppm (HSQC). In the HMBC spectrum, an interaction between CH_3 protons and the neighboring carbons C5 and C4 is visible but not with C3, which is 4 bonds away. Moreover, no nOe is observed between CH_3 and Ar-CH, confirming the 4-chloro regioisomer.

5-Methyl-4-chloro-2-trichloroacetyl-1*H*-pyrrole (**8**), HSQC NMR (DMSO-d_6):

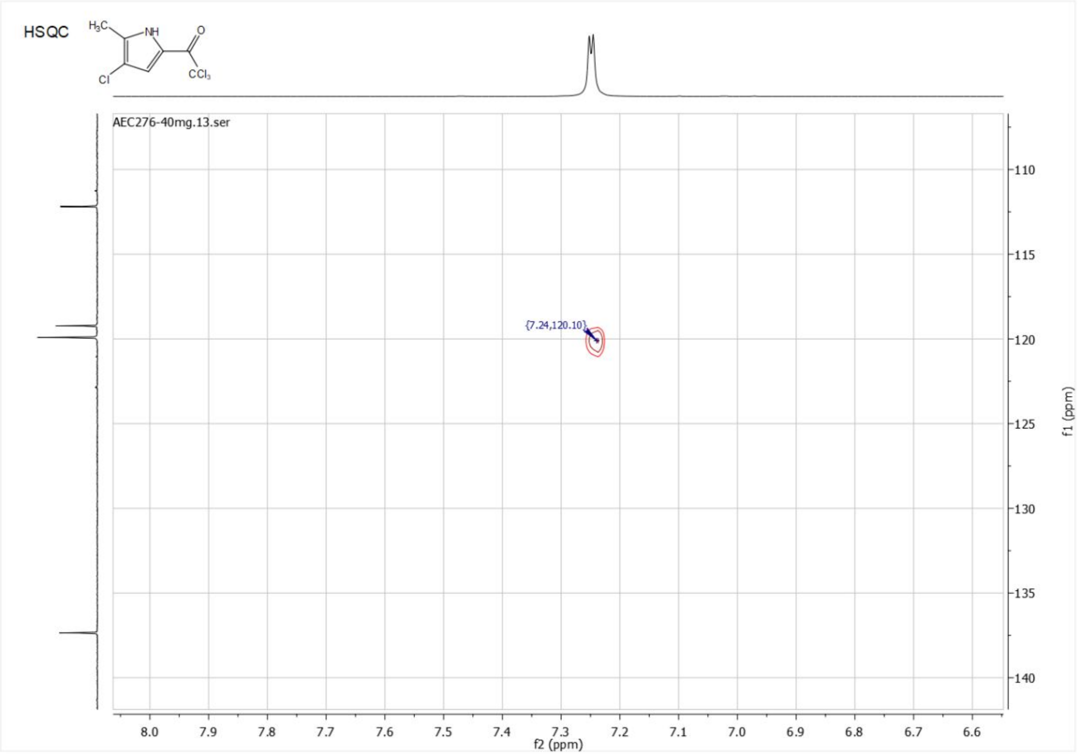

5-Methyl-4-chloro-2-trichloroacetyl-1*H*-pyrrole (**8**), HMBC NMR (DMSO-d_6):

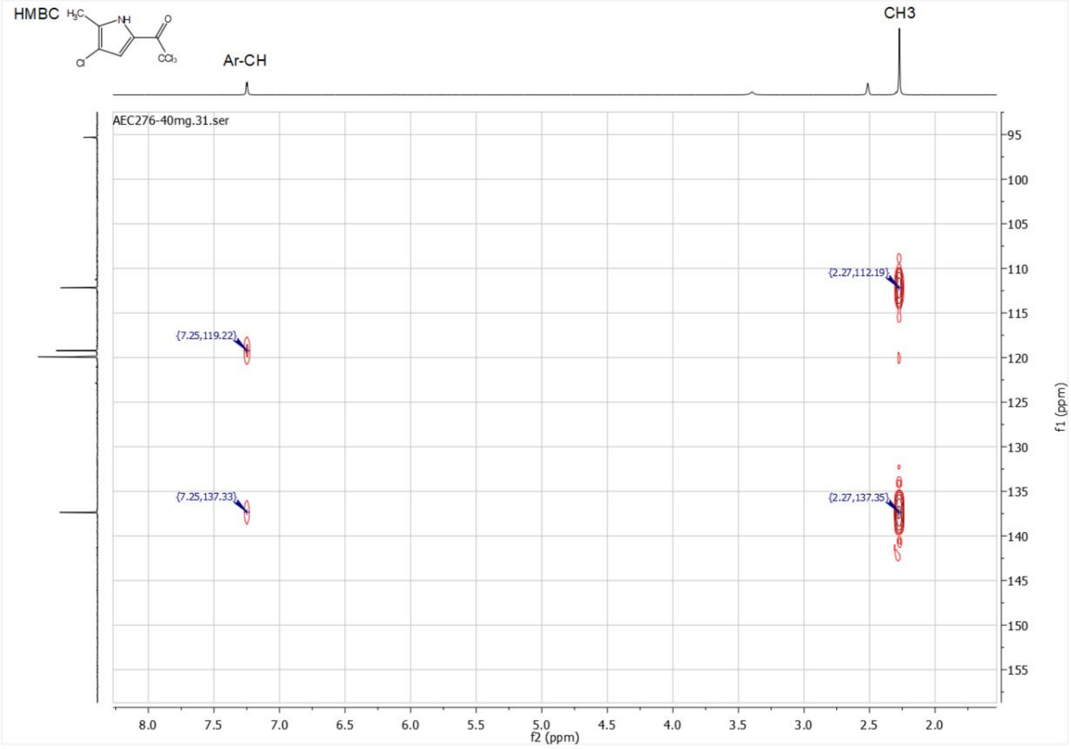

5-Methyl-4-chloro-2-trichloroacetyl-1*H*-pyrrole (**8**), NOESY NMR (DMSO-d_6):

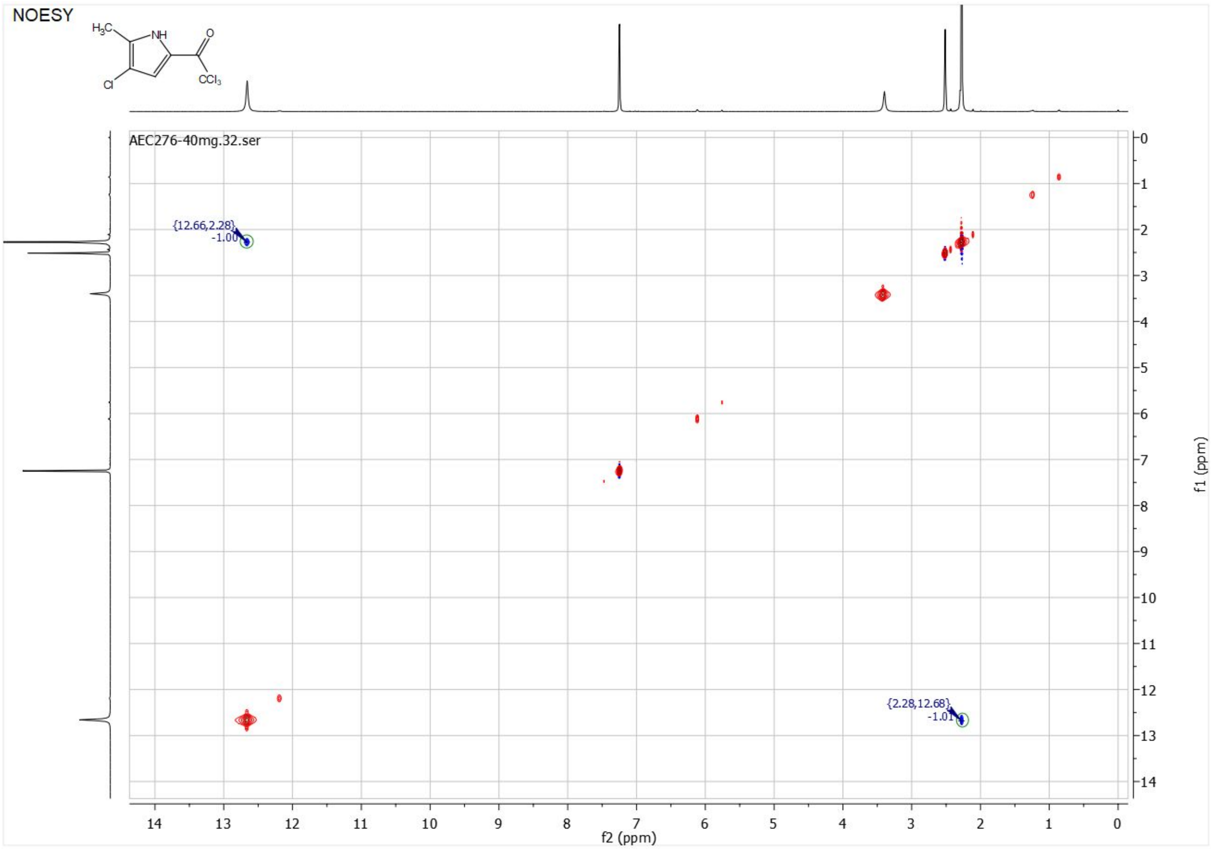

Ethyl 4-fluoro-5-methyl-1*H*-pyrrole-2-carboxylate (**10**), ^{1}H NMR (400 MHz, $CDCl_3$):

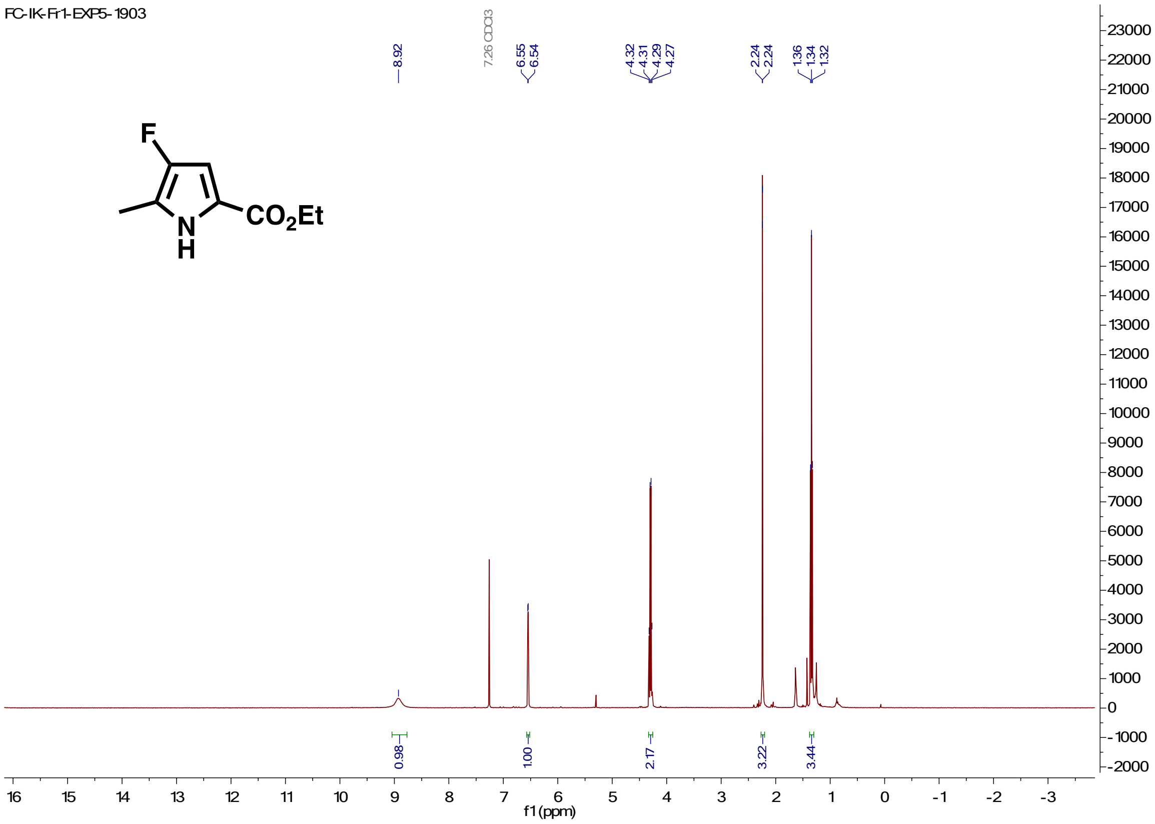

Ethyl 4-fluoro-5-methyl-1*H*-pyrrole-2-carboxylate (**10**), ^{13}C NMR (100 MHz, $CDCl_3$):

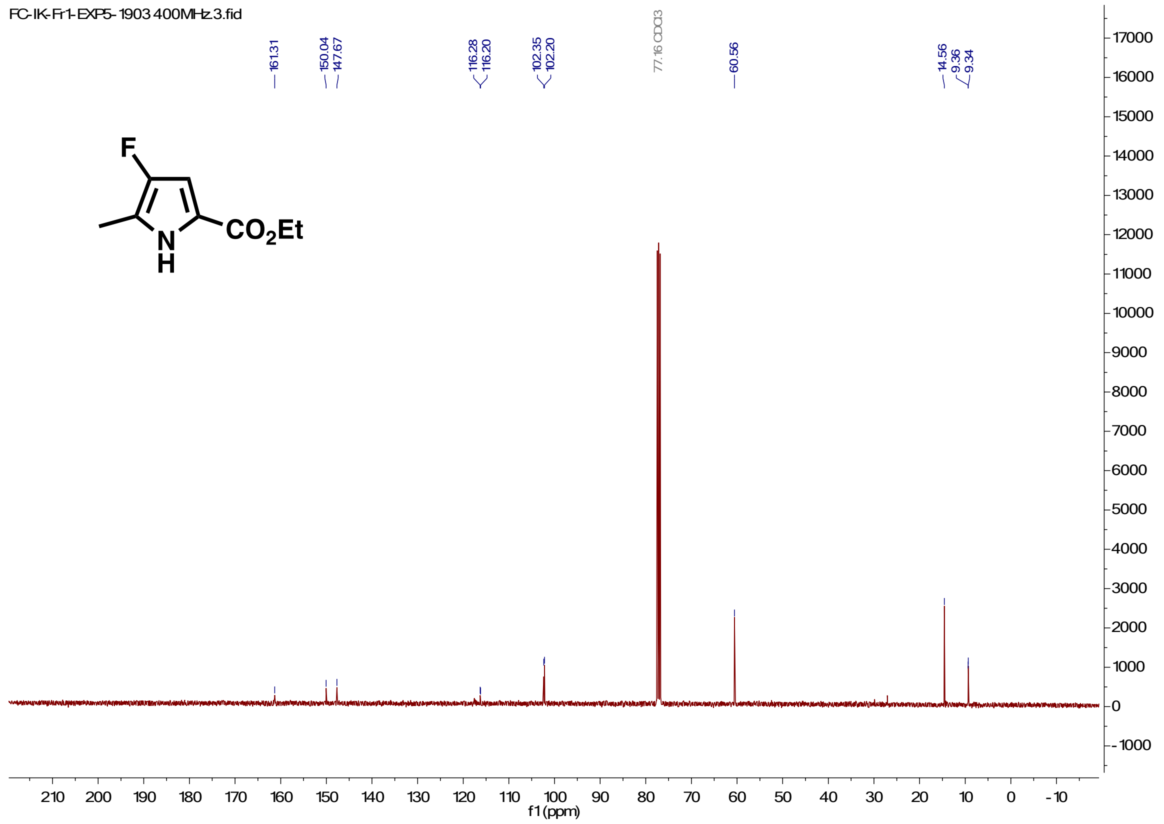

Ethyl 4-acetoxy-5-methyl-1*H*-pyrrole-2-carboxylate (**11**), ^{1}H NMR (400 MHz, $CDCl_3$):

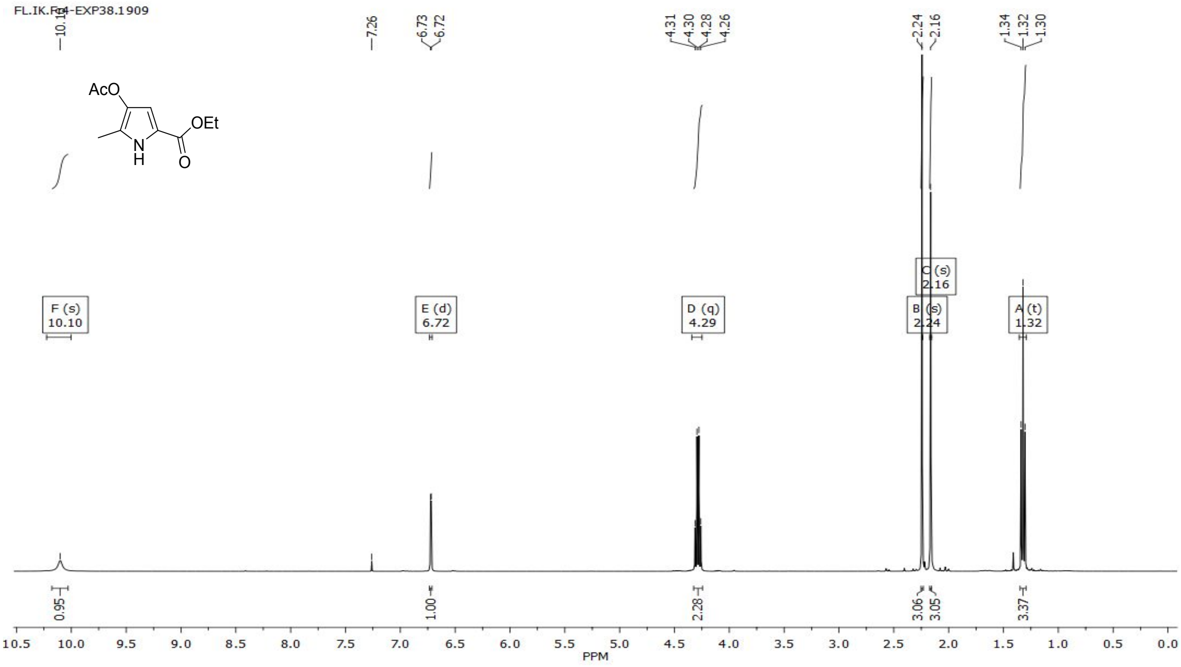

Ethyl 4-acetoxy-5-methyl-1*H*-pyrrole-2-carboxylate (**11**), ^{13}C NMR (100 MHz, $CDCl_3$):

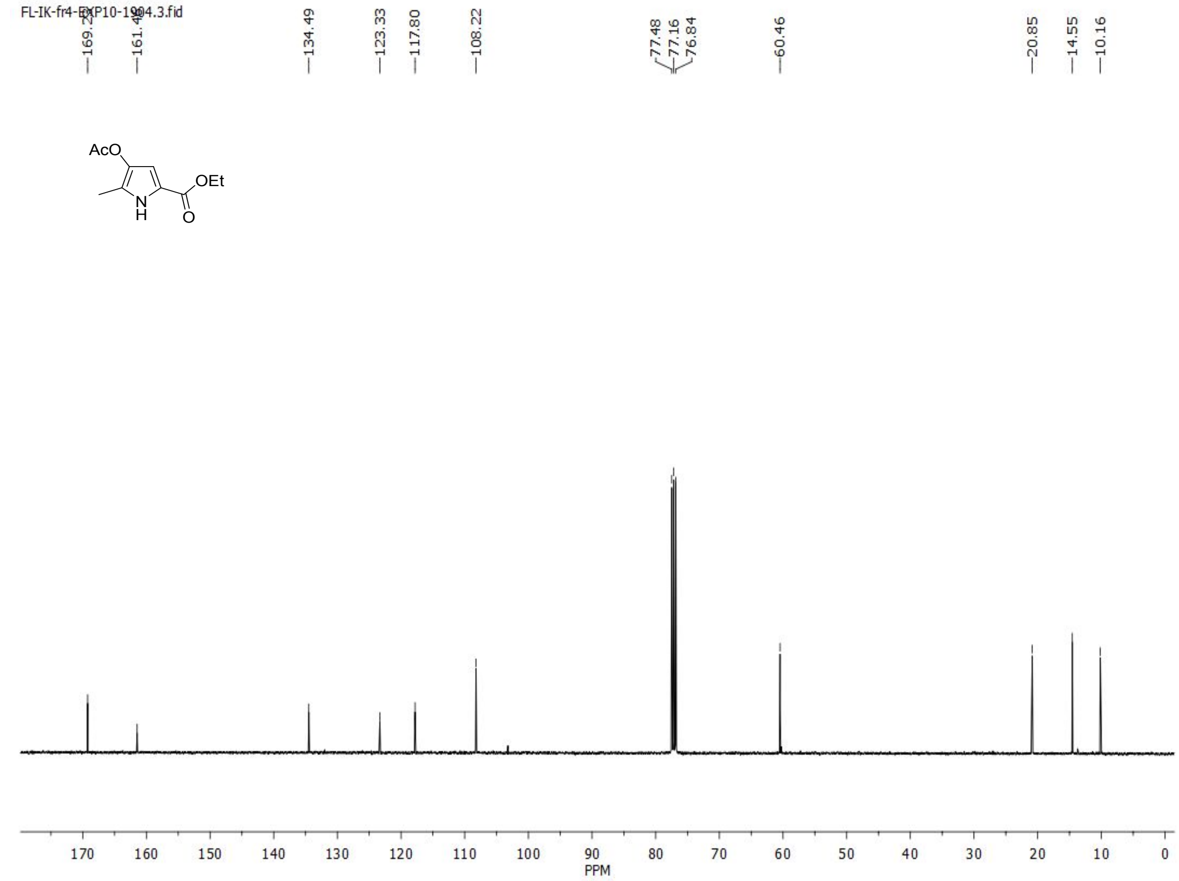

4-Fluoro-5-methyl-1*H*-pyrrole-2-carboxylic acid (**12**), ^{1}H NMR (400 MHz, DMSO-d_6):

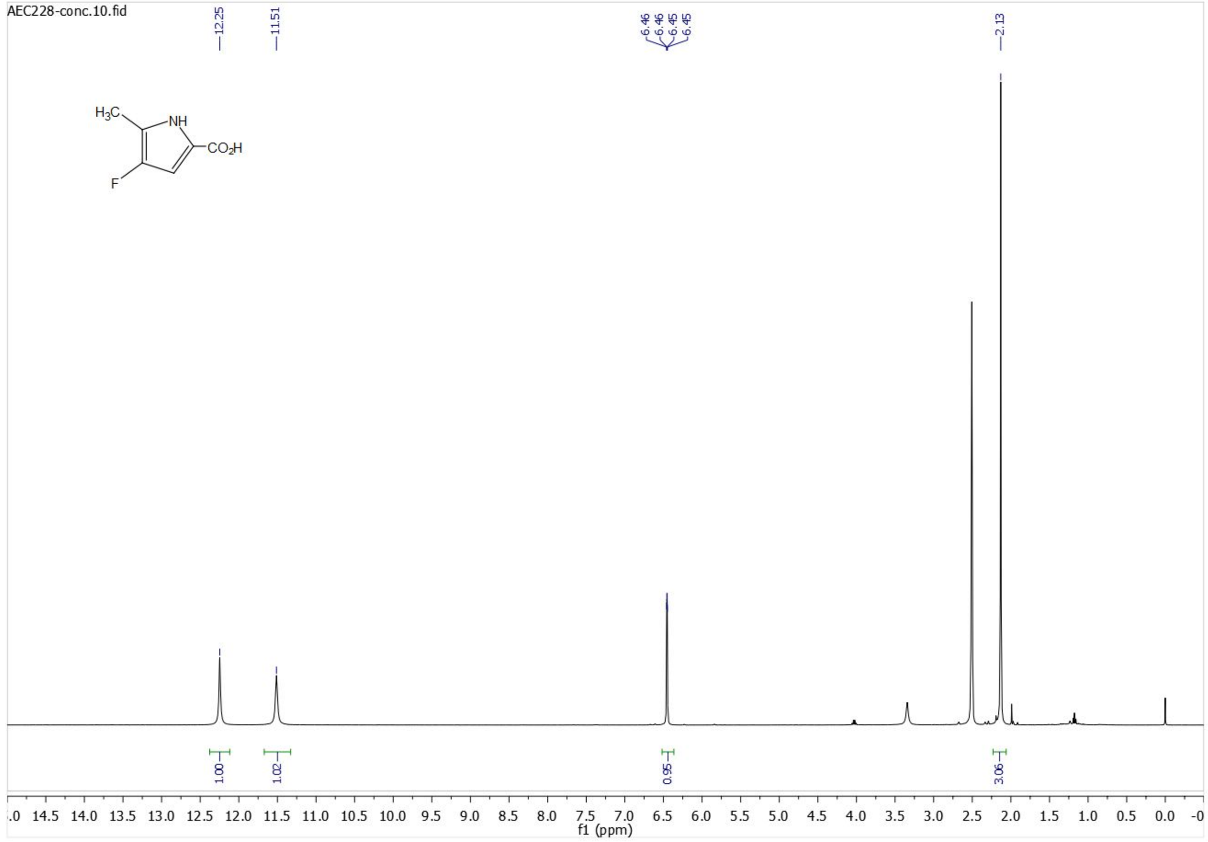

4-Fluoro-5-methyl-1*H*-pyrrole-2-carboxylic acid (**12**), ^{13}C NMR (100 MHz, DMSO-d_6):

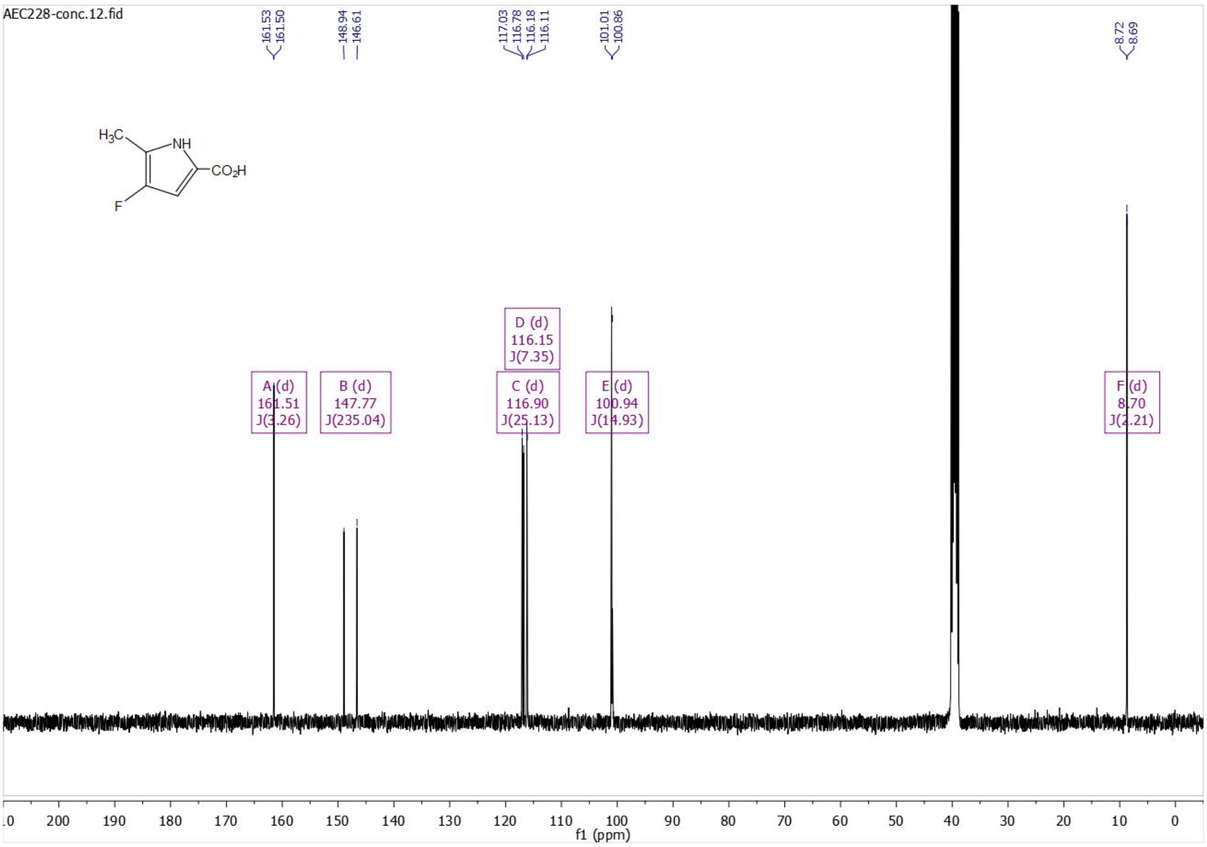

4-Fluoro-5-methyl-1*H*-pyrrole-2-carbonyl chloride (**13**), ^{1}H NMR (400 MHz, $CDCl_3$):

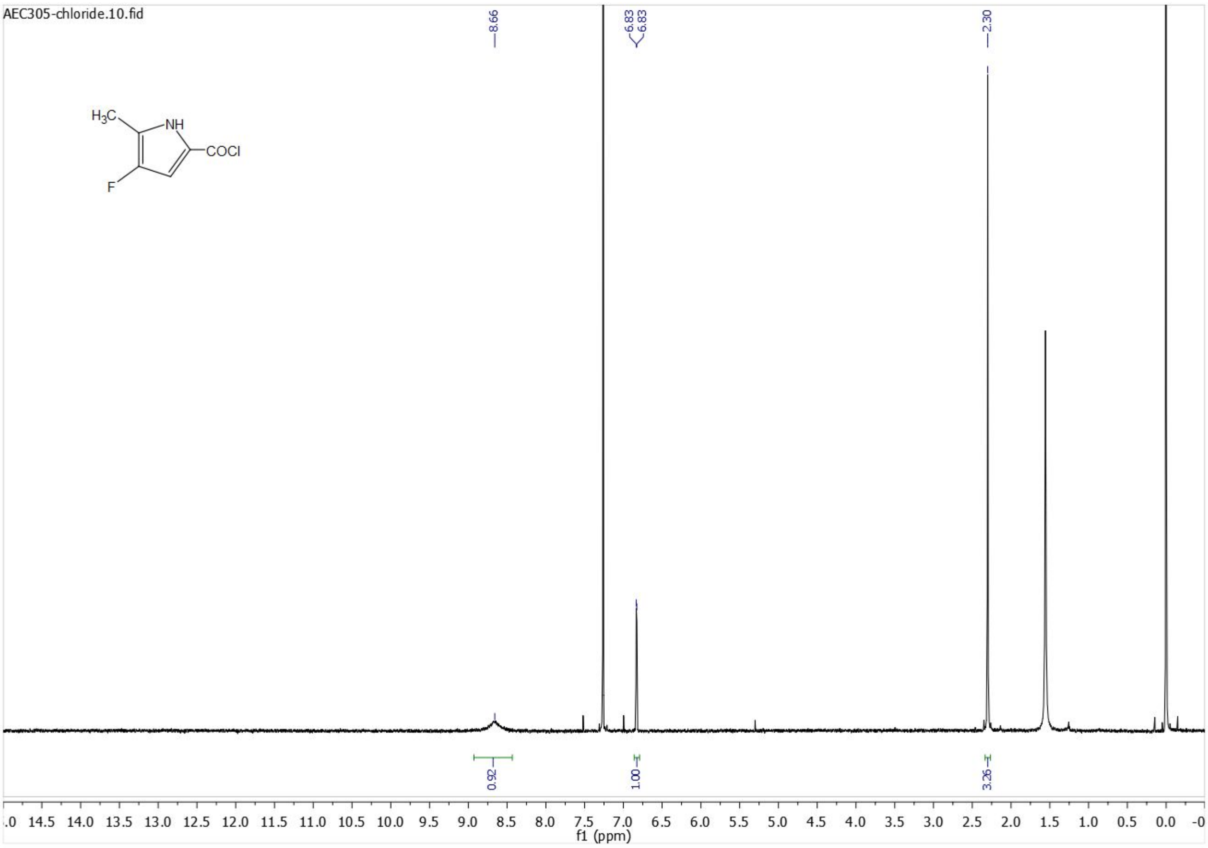

Ethyl 3-fluoro-5-formyl-1*H*-pyrrole-2-carboxylate (**15**), ^{1}H NMR (400 MHz, $CDCl_3$):

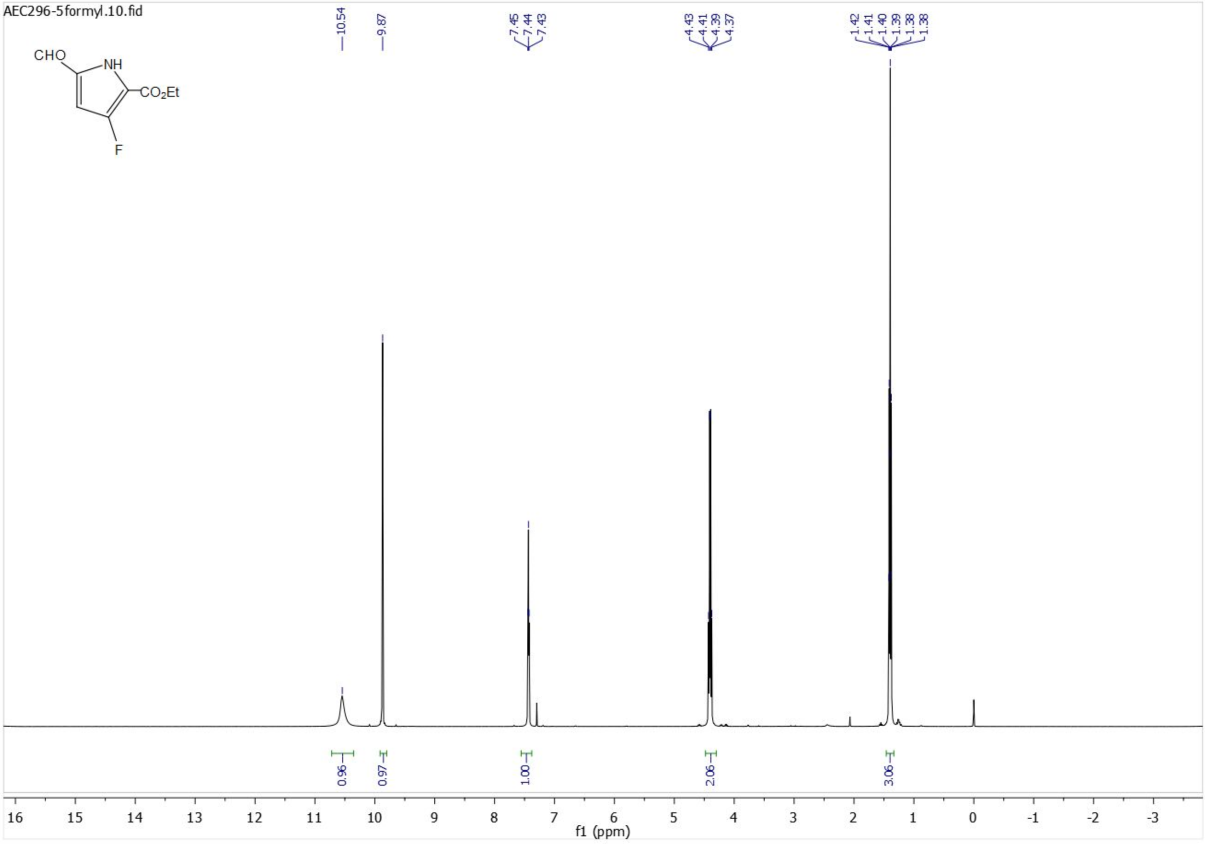

Ethyl 3-fluoro-5-formyl-1*H*-pyrrole-2-carboxylate (**15**), ^{13}C NMR (100 MHz, $CDCl_3$):

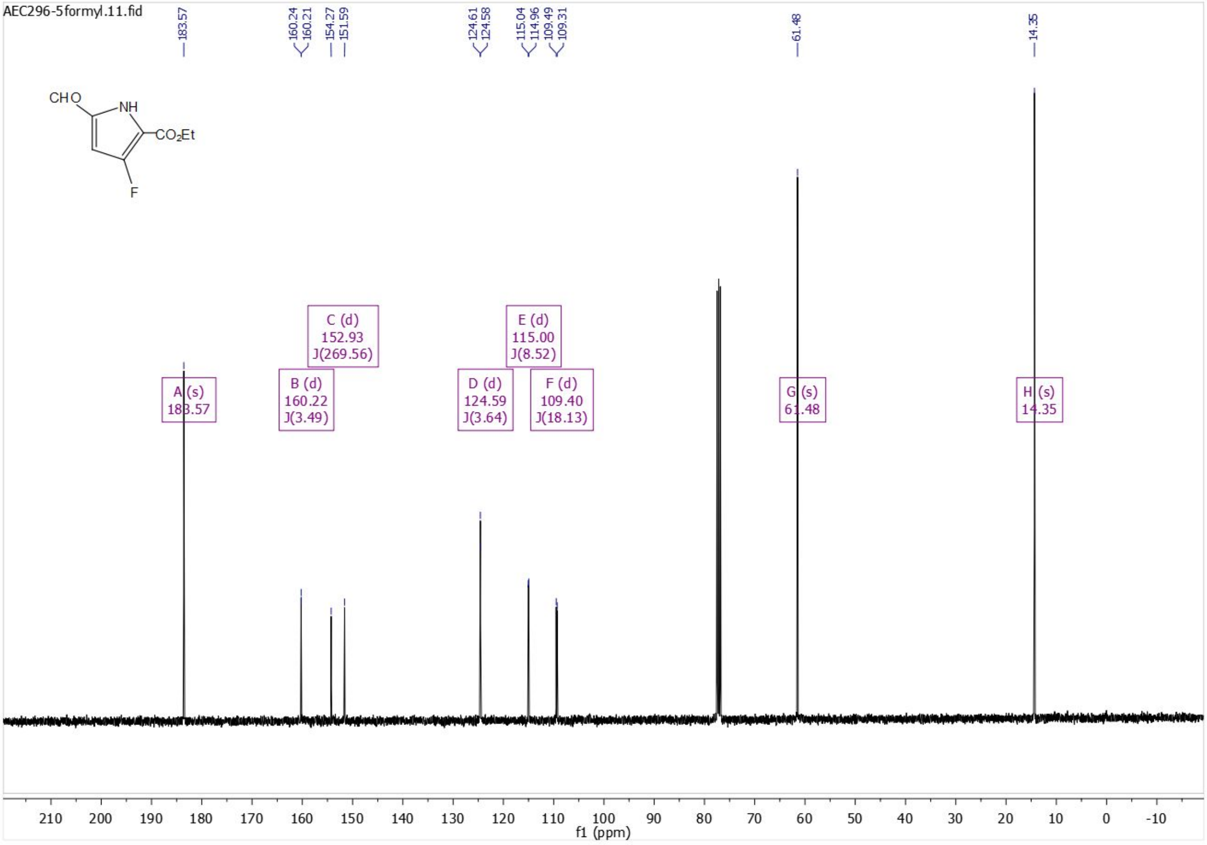

Ethyl 3-fluoro-4-formyl-1*H*-pyrrole-2-carboxylate (**16**), ^{1}H NMR (400 MHz, $CDCl_3$):

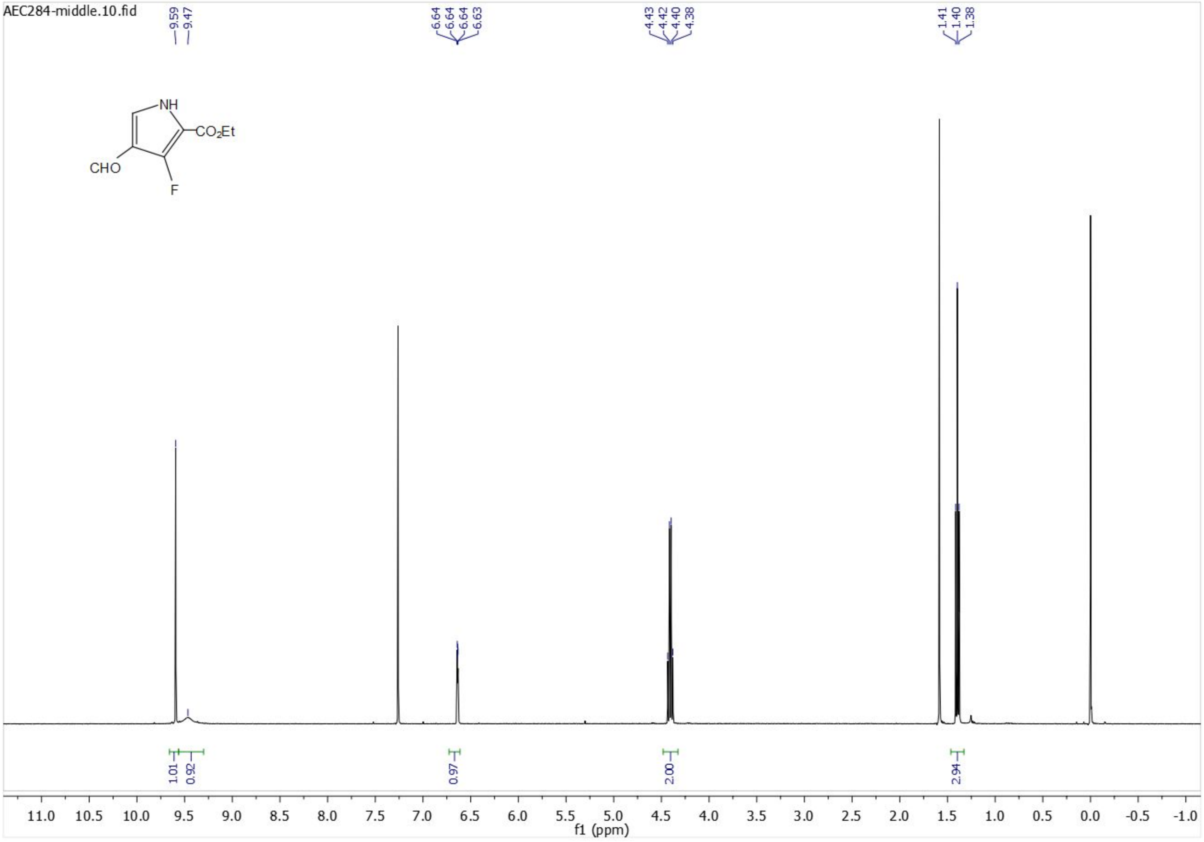

Ethyl 3-fluoro-4-formyl-1*H*-pyrrole-2-carboxylate (**16**), ^{13}C NMR (100 MHz, $CDCl_3$):

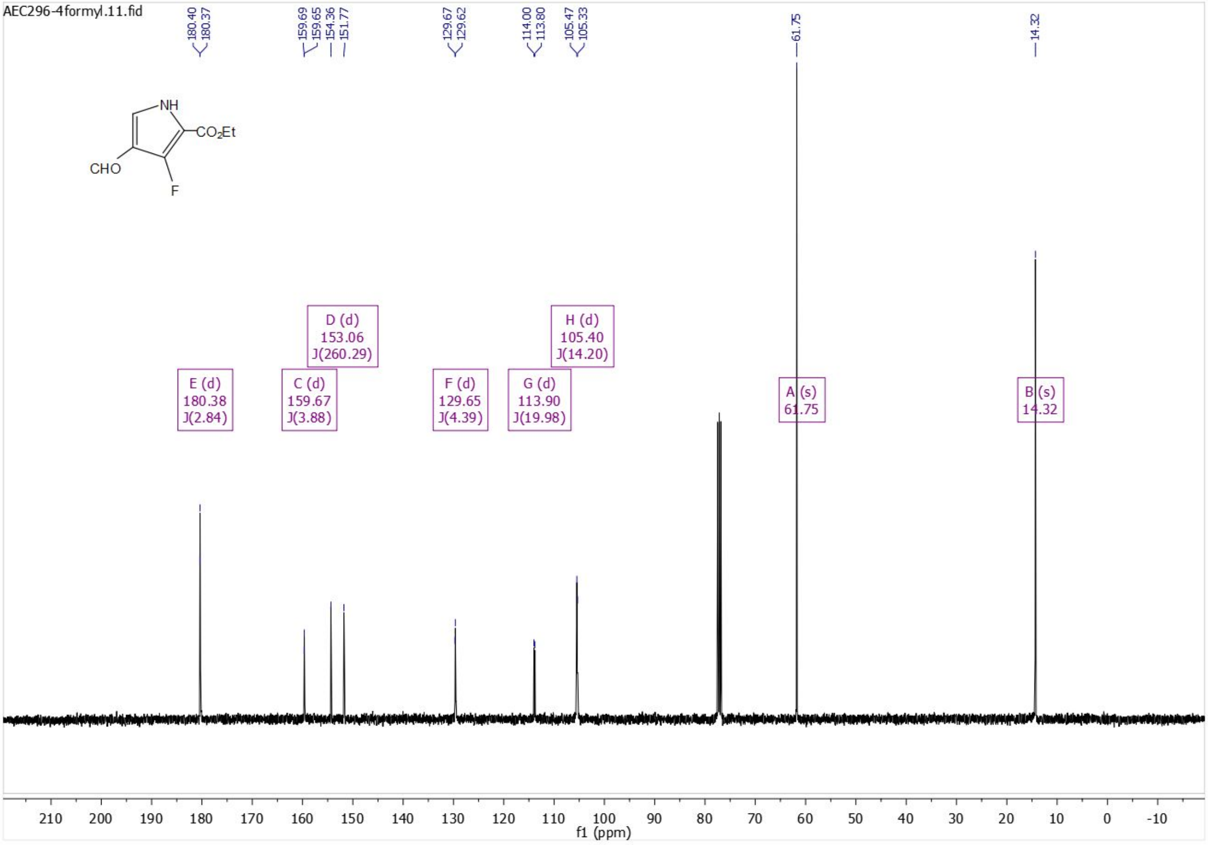

Ethyl 3-fluoro-5-methyl-1*H*-pyrrole-2-carboxylate (**17**), ^{1}H NMR (400 MHz, $CDCl_3$):

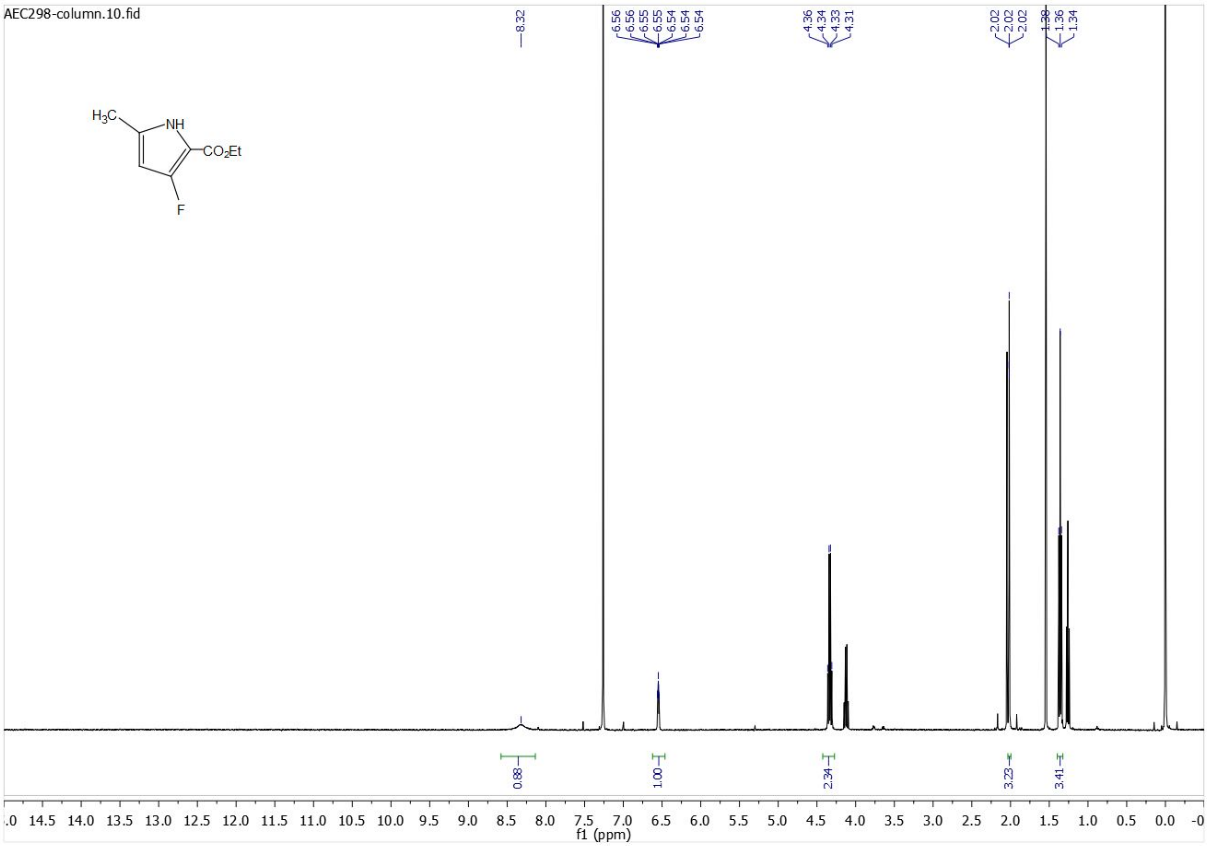

3-Fluoro-5-methyl-1*H*-pyrrole-2-carboxylic acid (**18**), ^{1}H NMR (400 MHz, DMSO-d_6):

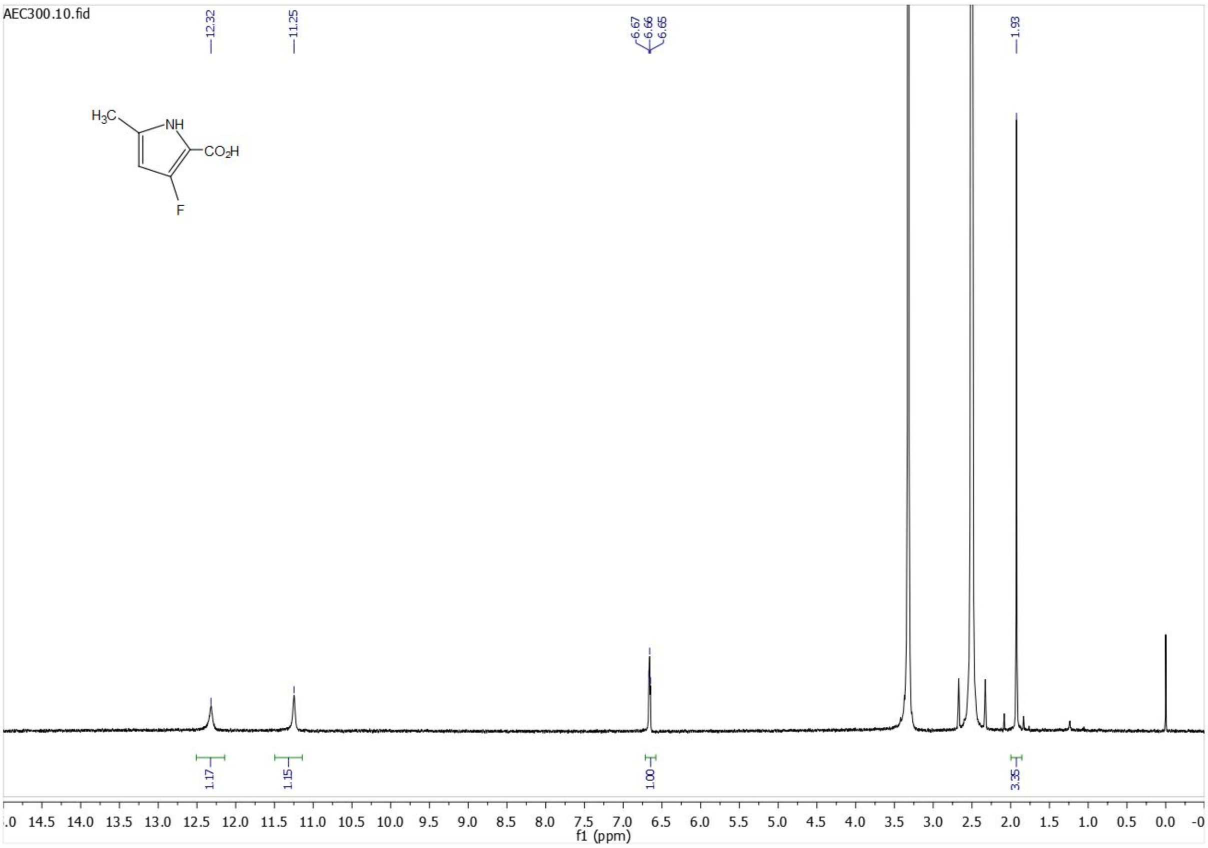

3-Fluoro-5-methyl-1*H*-pyrrole-2-carboxylic acid (**18**), ^{13}C NMR (100 MHz, DMSO-d_6):

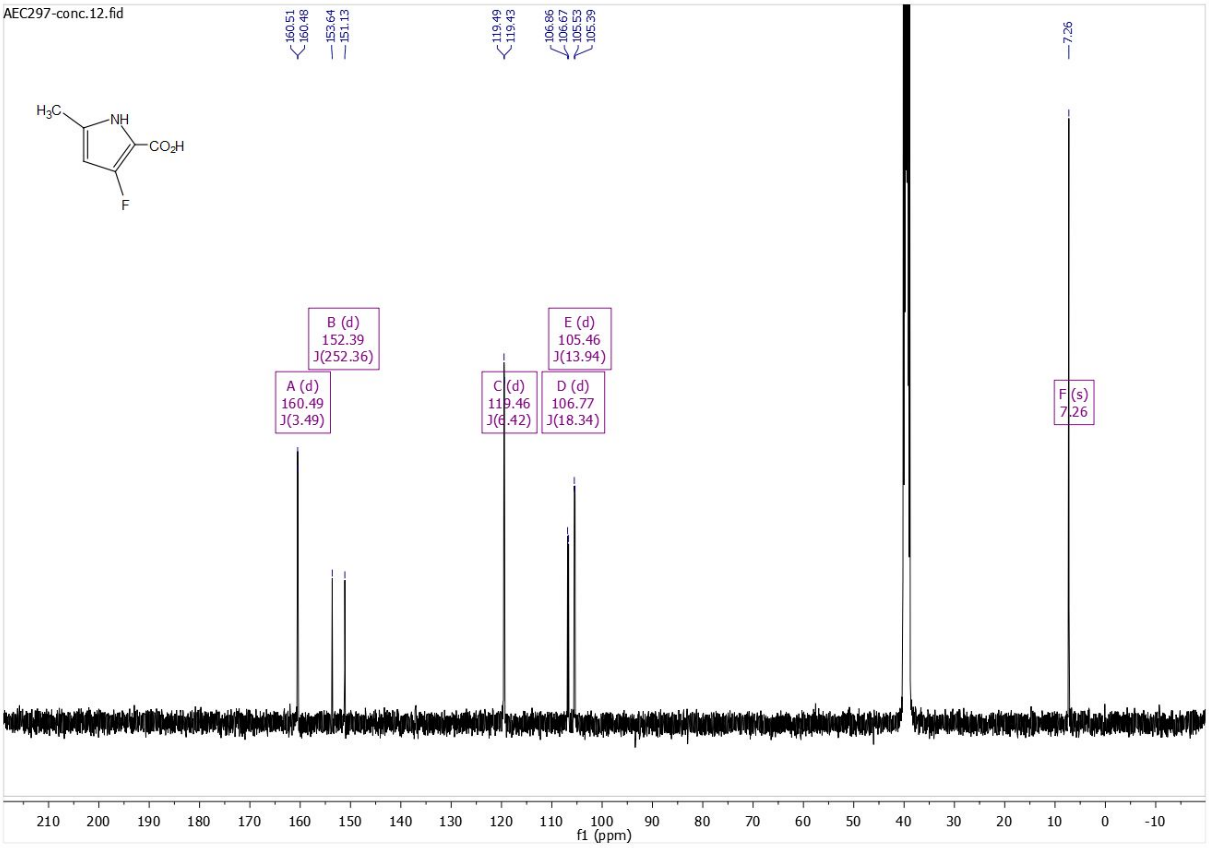

3-Fluoro-5-methyl-1*H*-pyrrole-2-carbonyl chloride (**19**), ^{1}H NMR (400 MHz, $CDCl_3$):

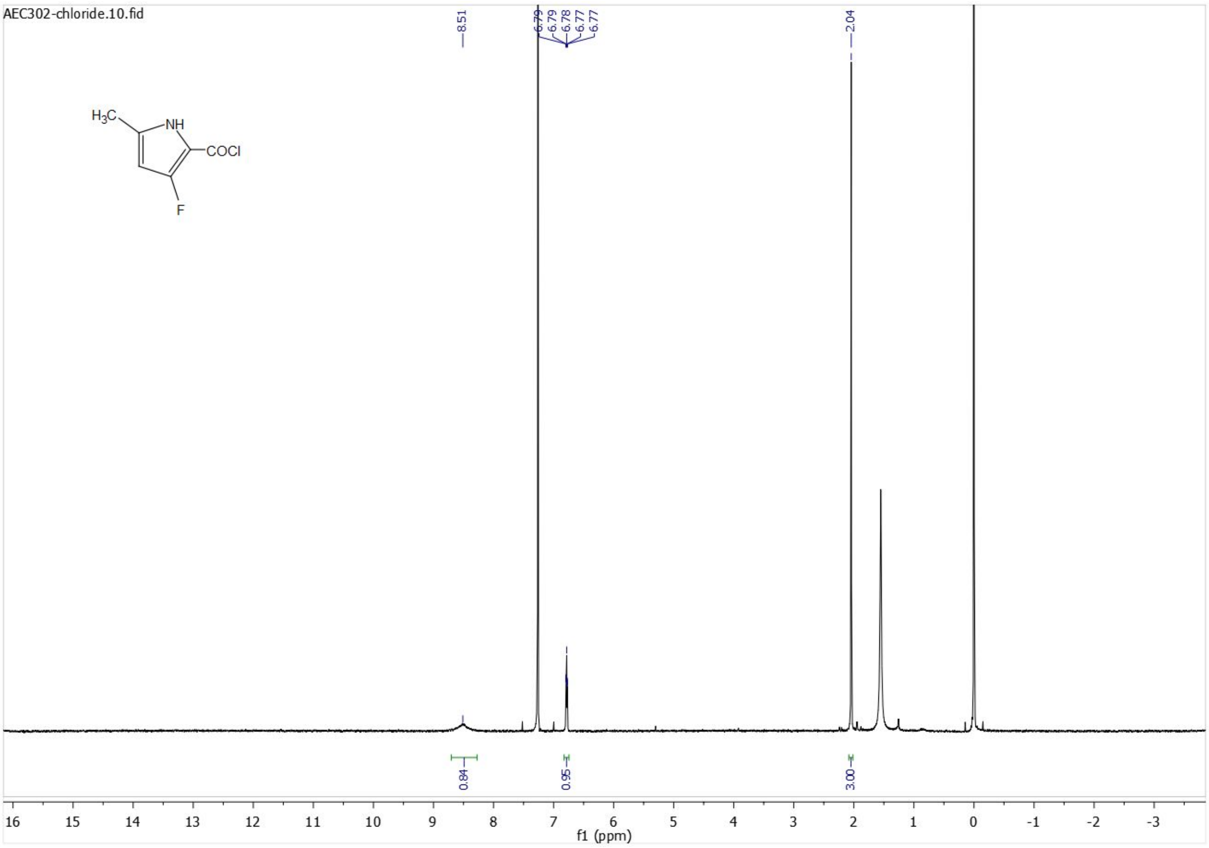

Ethyl 3,4-dichloro-5-azidomethyl-1*H*-pyrrole-2-carboxylate (**22**), ^{1}H NMR (400 MHz, $CDCl_3$):

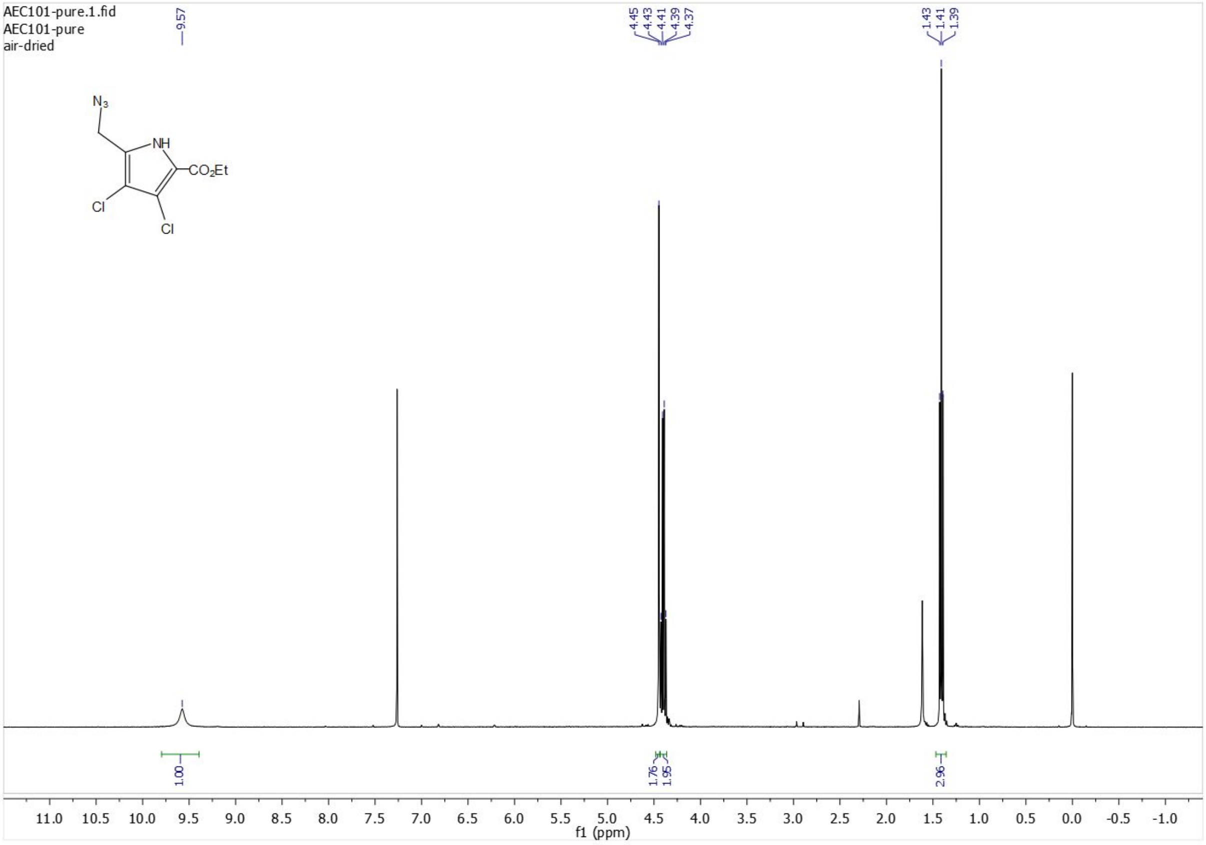

Ethyl 3,4-dichloro-5-azidomethyl-1*H*-pyrrole-2-carboxylate (**22**), ^{13}C NMR (100 MHz, $CDCl_3$):

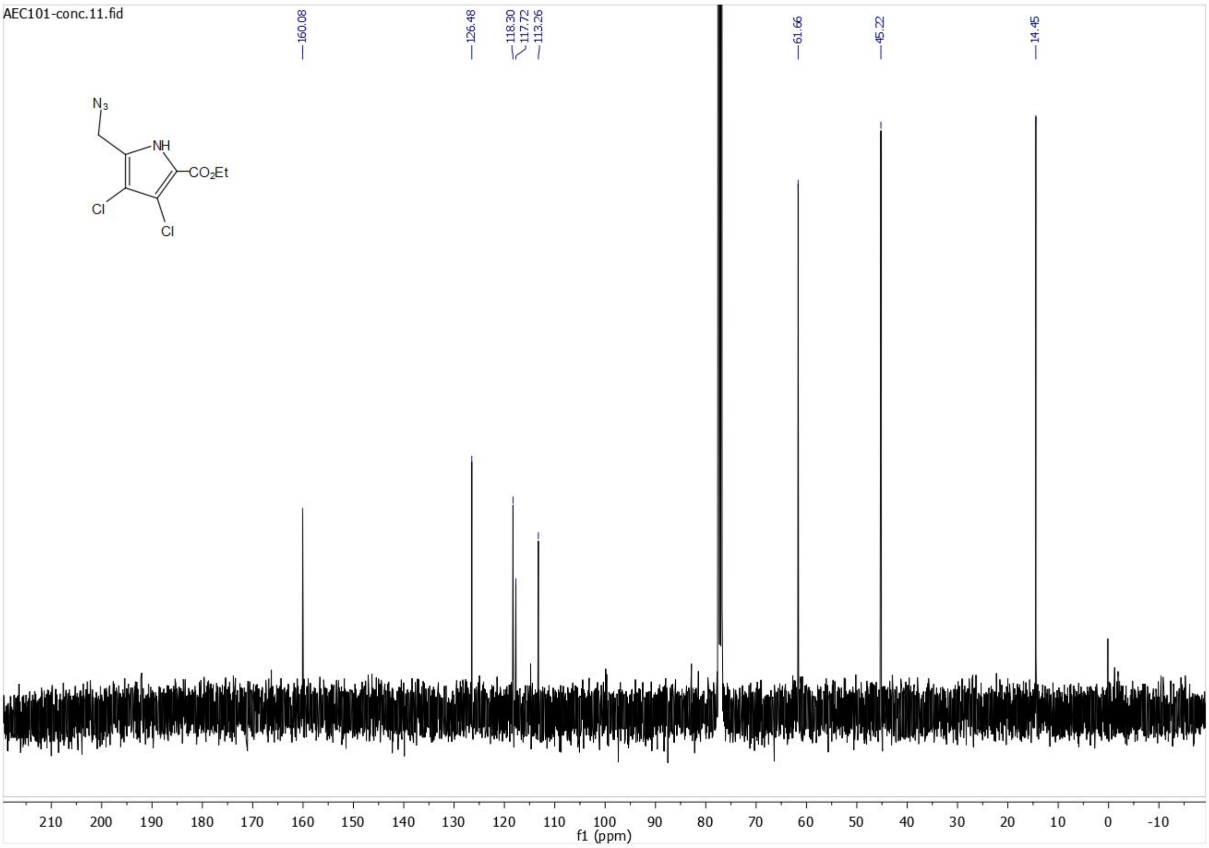

Ethyl 3,4-dichloro-5-aminomethyl-1*H*-pyrrole-2-carboxylate (**23**), ^{1}H NMR (400 MHz, $CDCl_3$):

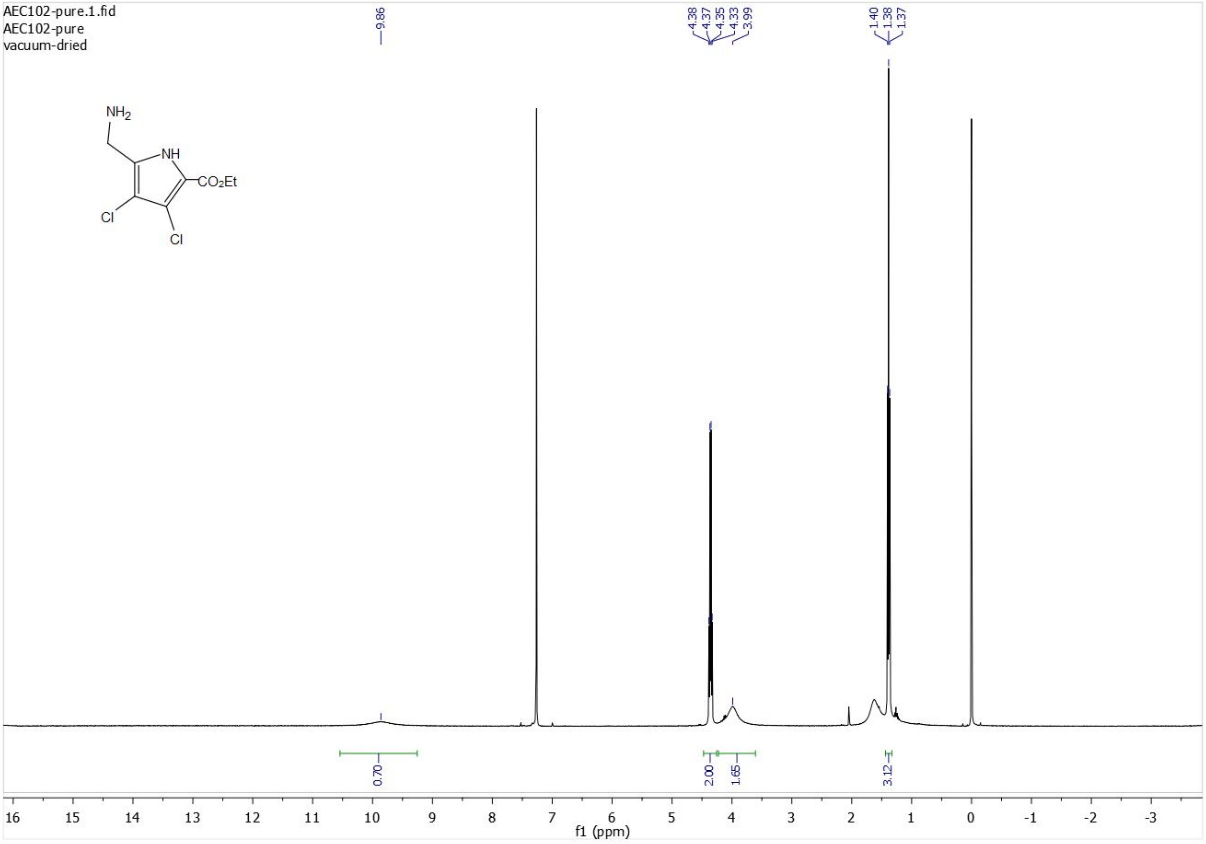

Ethyl 3,4-dichloro-5-aminomethyl-1*H*-pyrrole-2-carboxylate (**23**), ^{13}C NMR (100 MHz, $CDCl_3$):

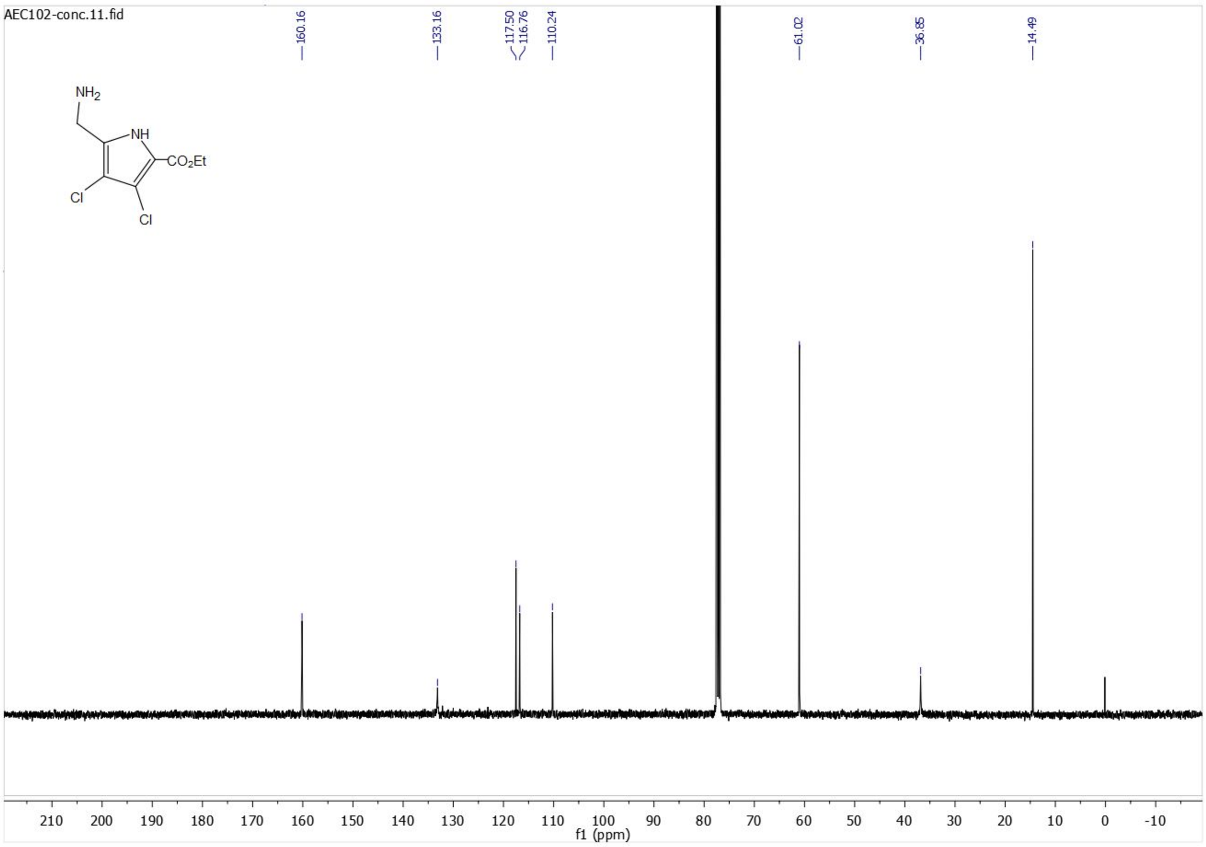

3,4-dichloro-5-aminomethyl-1*H*-pyrrole-2-carboxylic acid (**24**), ^{1}H NMR (400 MHz, DMSO-d_6):

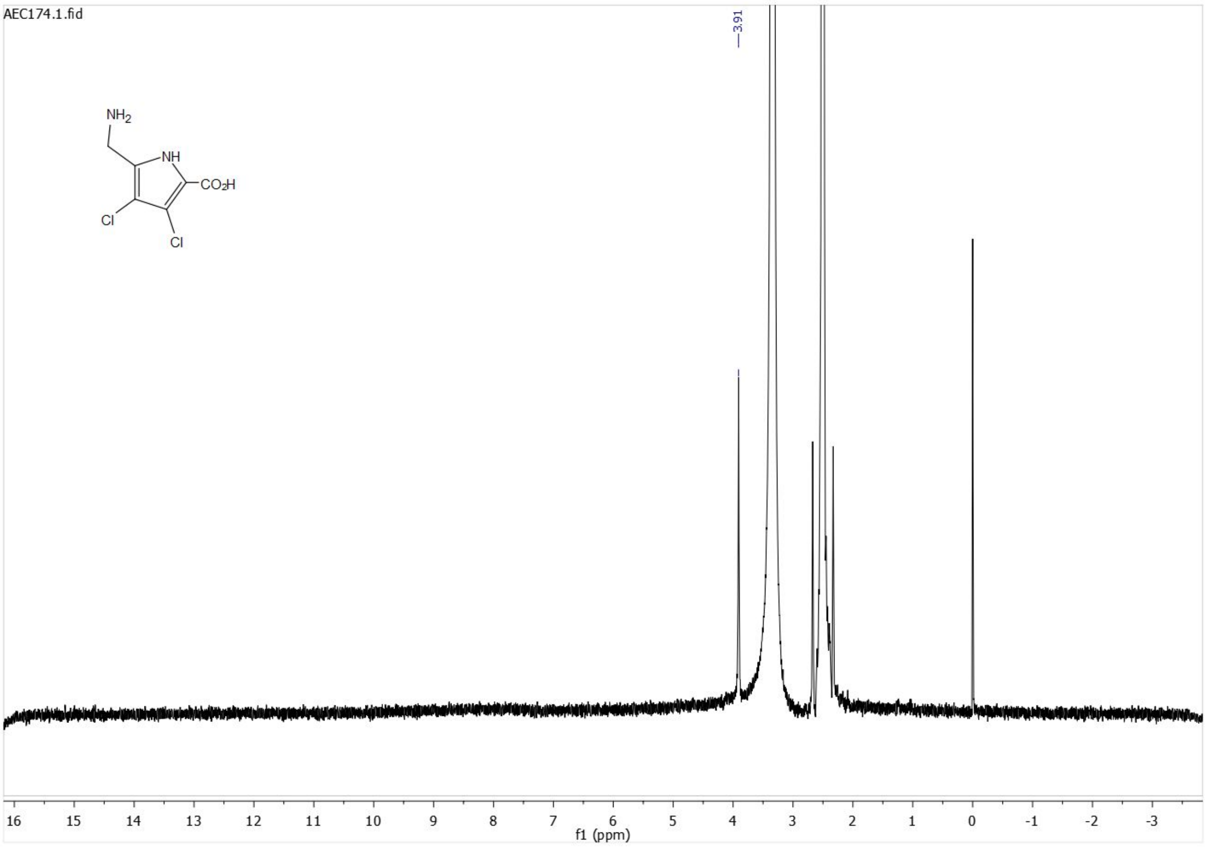

3,4-Dichloro-5-phthalimidomethyl-1*H*-pyrrole-2-carboxylic acid (**25**), ^{1}H NMR (400 MHz, DMSO-d_6):

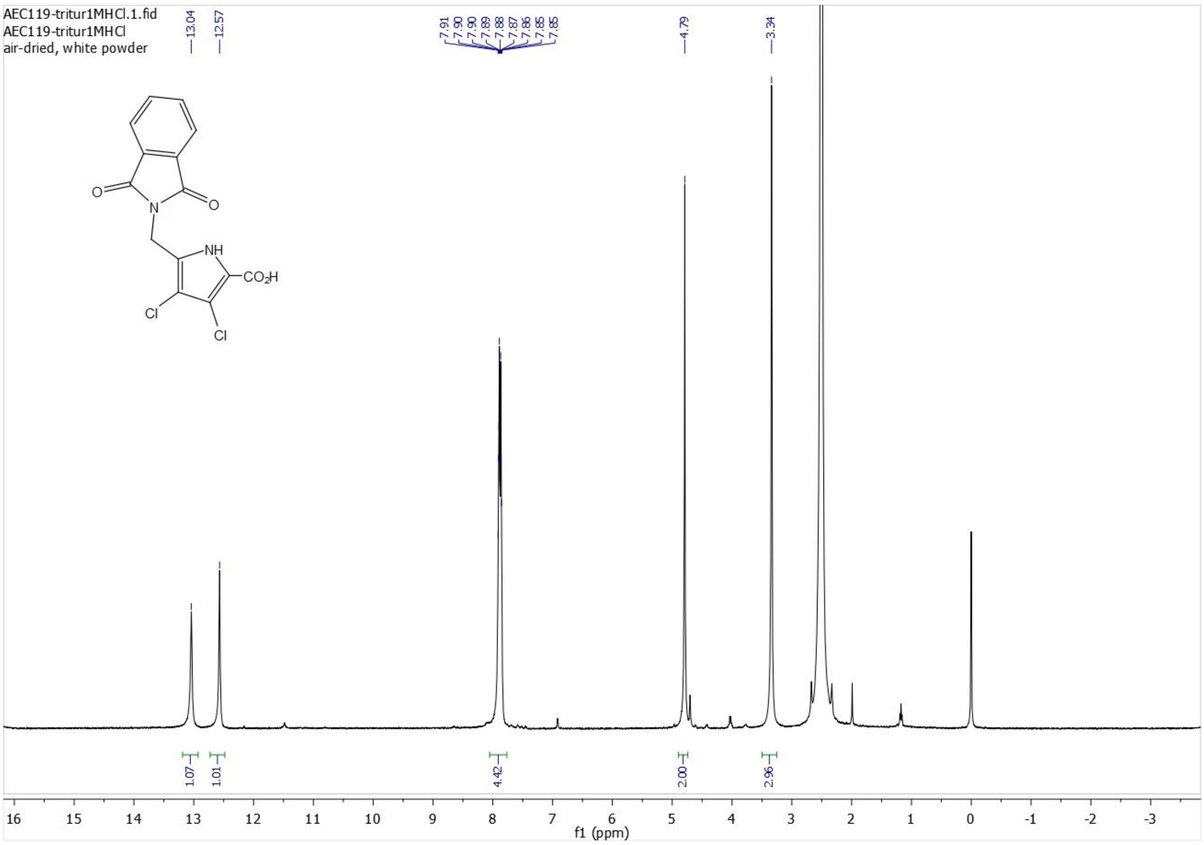

3,4-Dichloro-5-phthalimidomethyl-1*H*-pyrrole-2-carboxylic acid (**25**), ^{13}C NMR (100 MHz, DMSO-d_6):

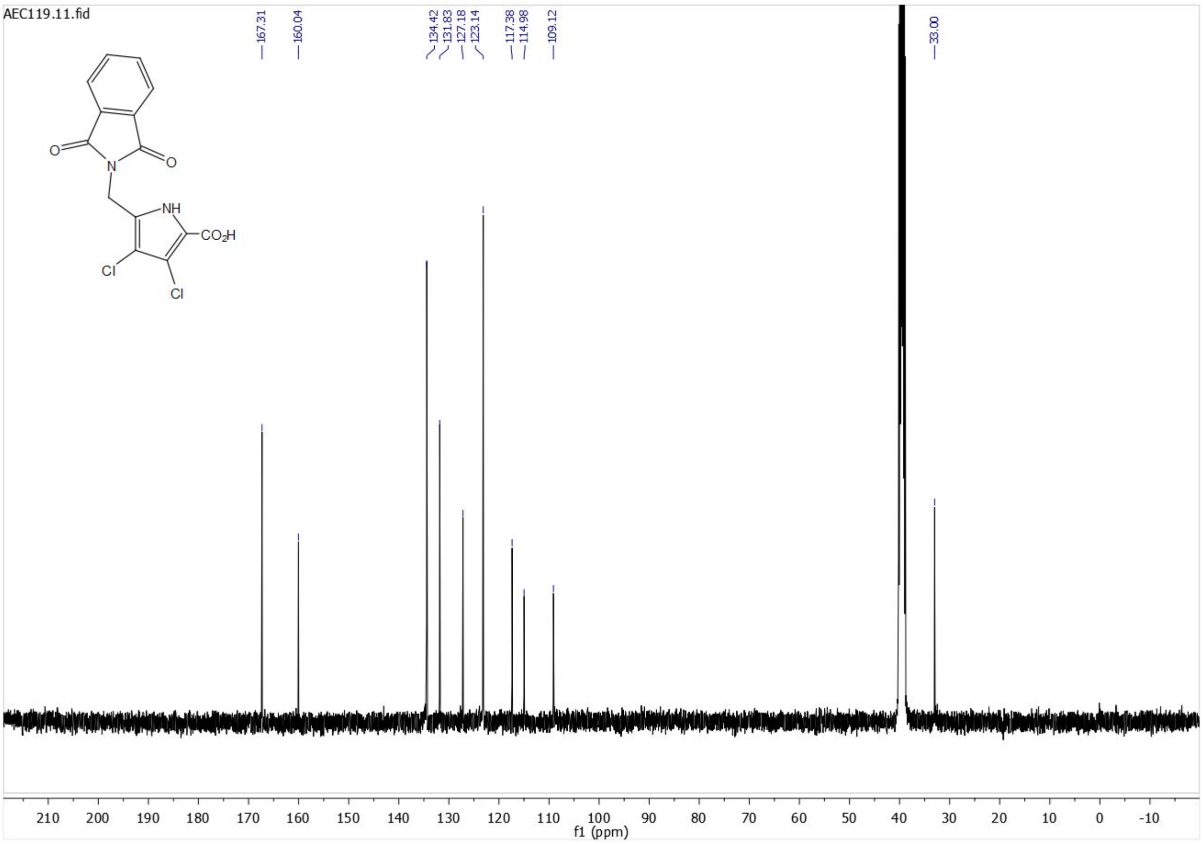

Compound **26**, ^{1}H NMR (400 MHz, DMSO-d_6):

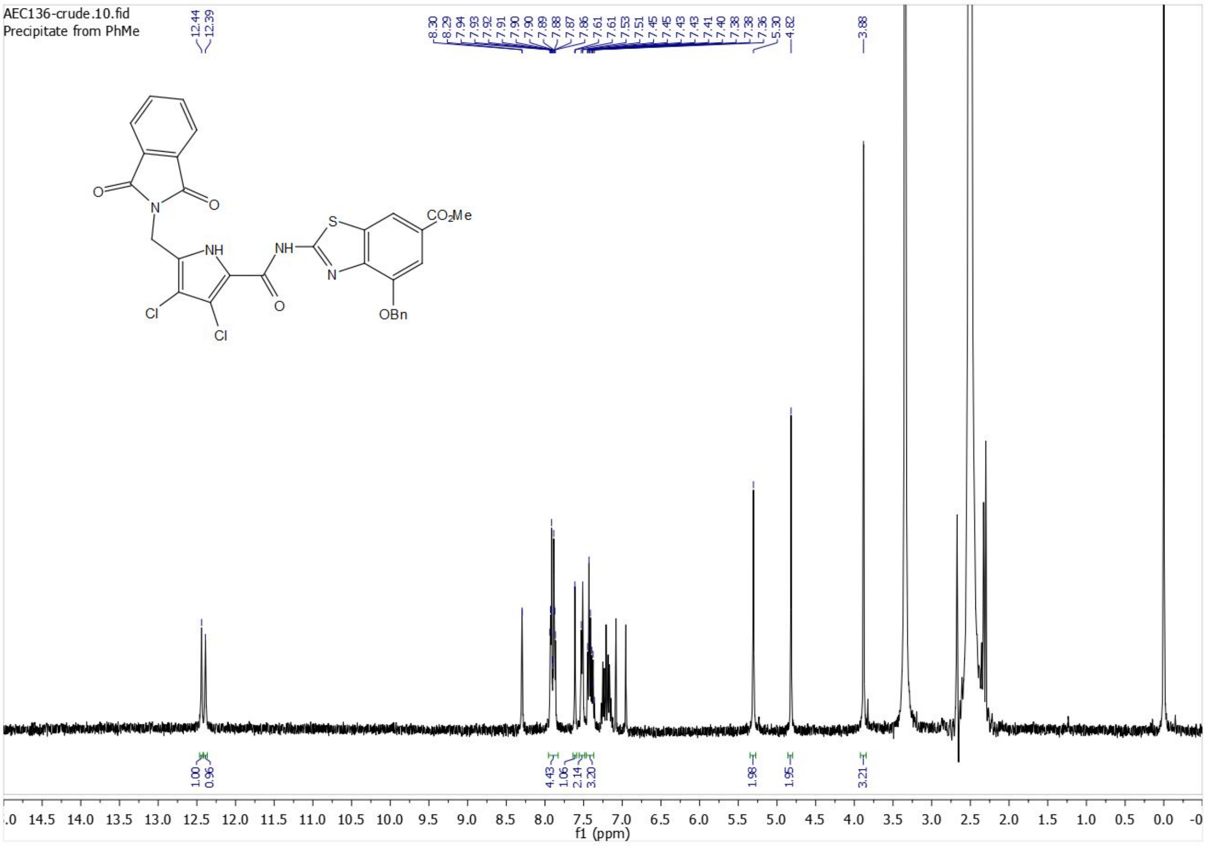

Compound **27**, ^{1}H NMR (400 MHz, DMSO-d_6) of the reaction mixture aliquot, containing EtOH:

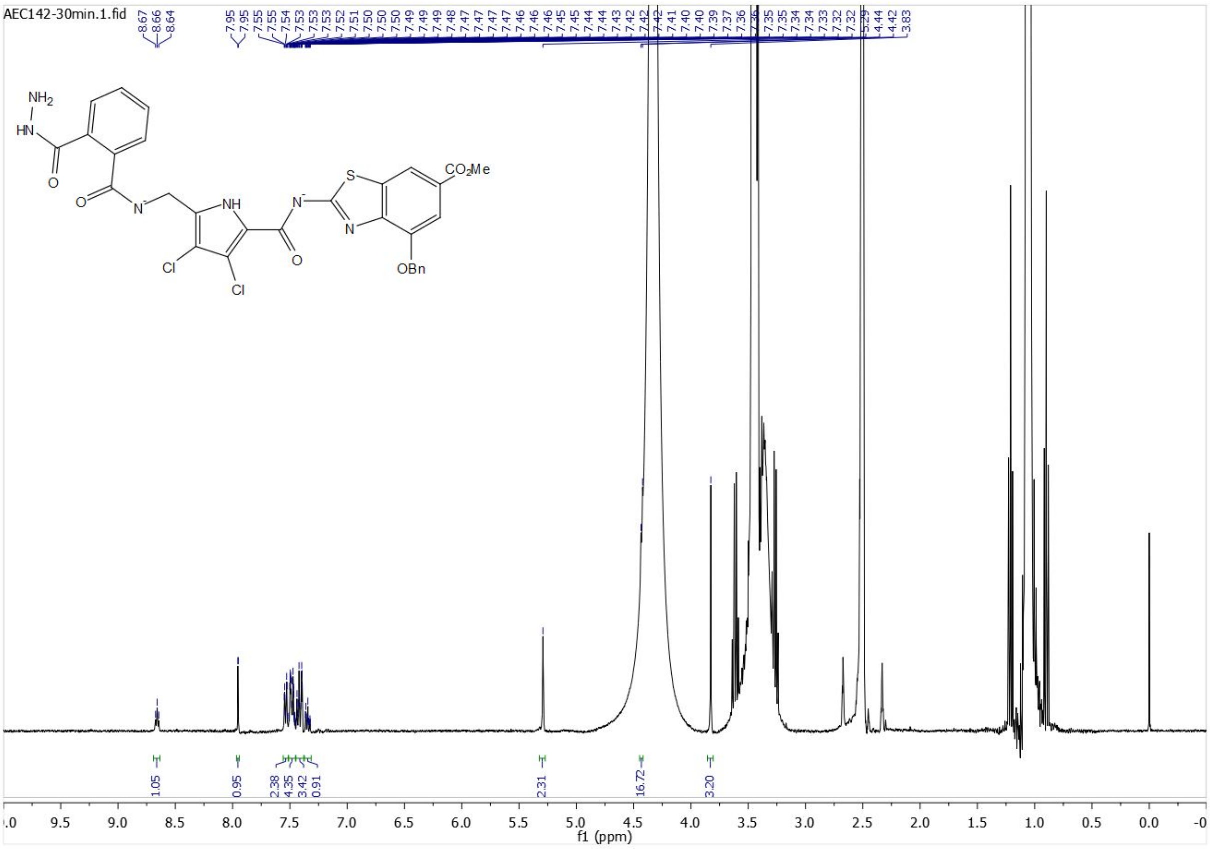

Reprotonated compound **27**, ^{1}H NMR (400 MHz, DMSO-d_6):

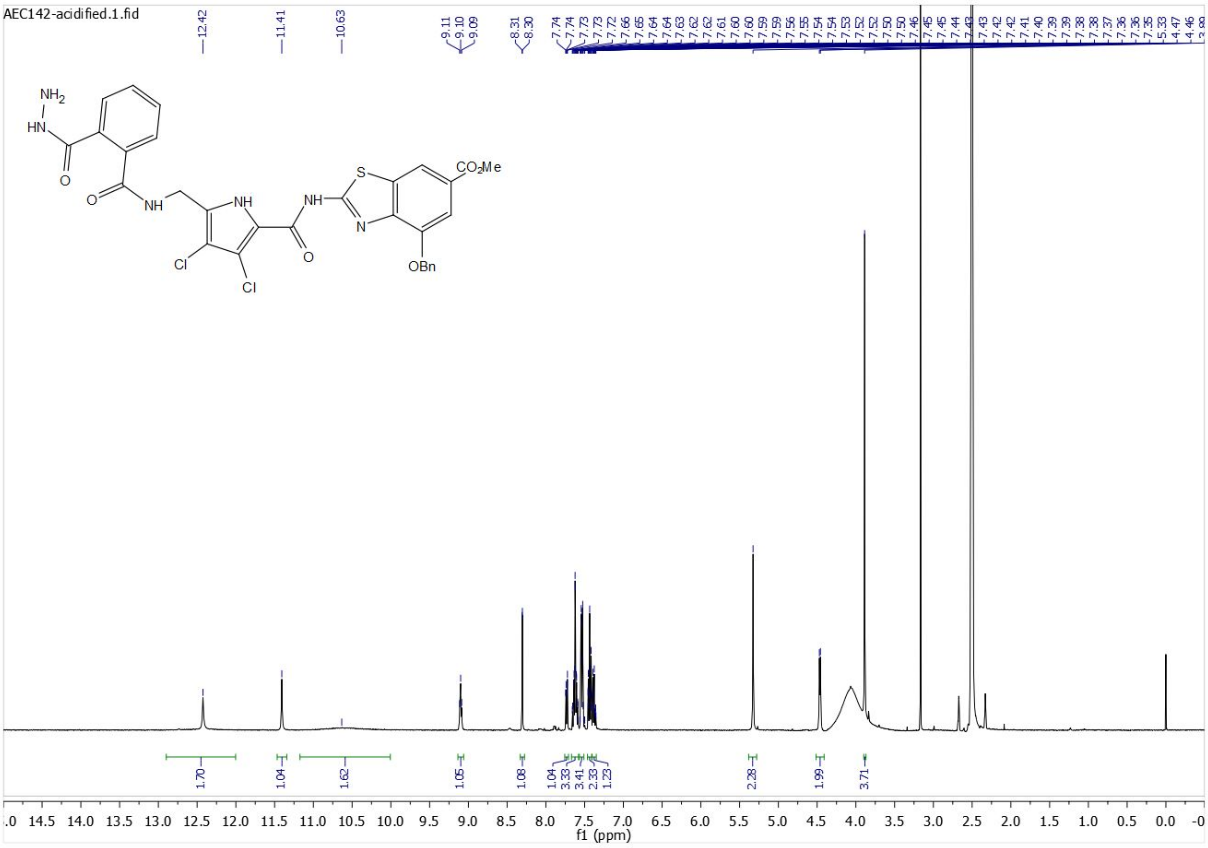

Compound **28**, ^{1}H NMR (400 MHz, DMSO-d_6):

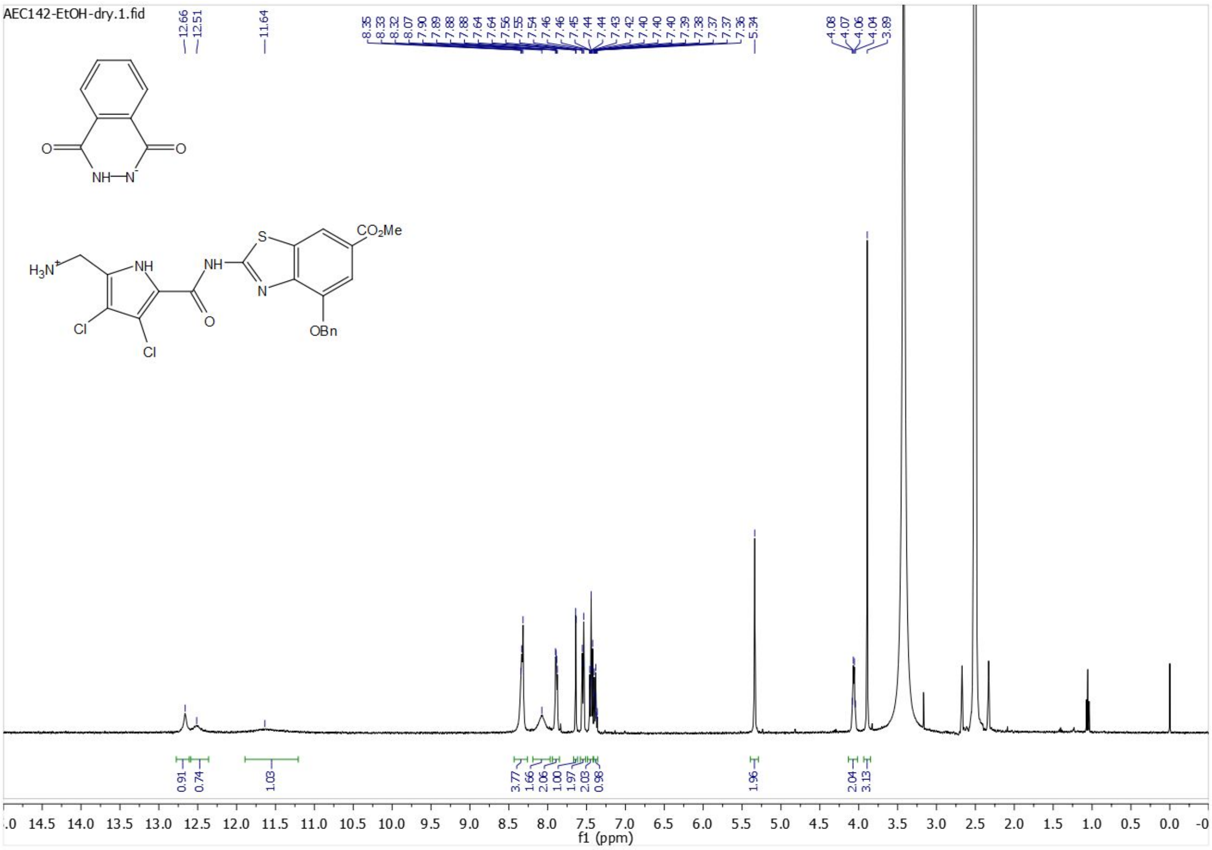

Compound **5·HCl**, ^{1}H NMR (400 MHz, DMSO-d_6):

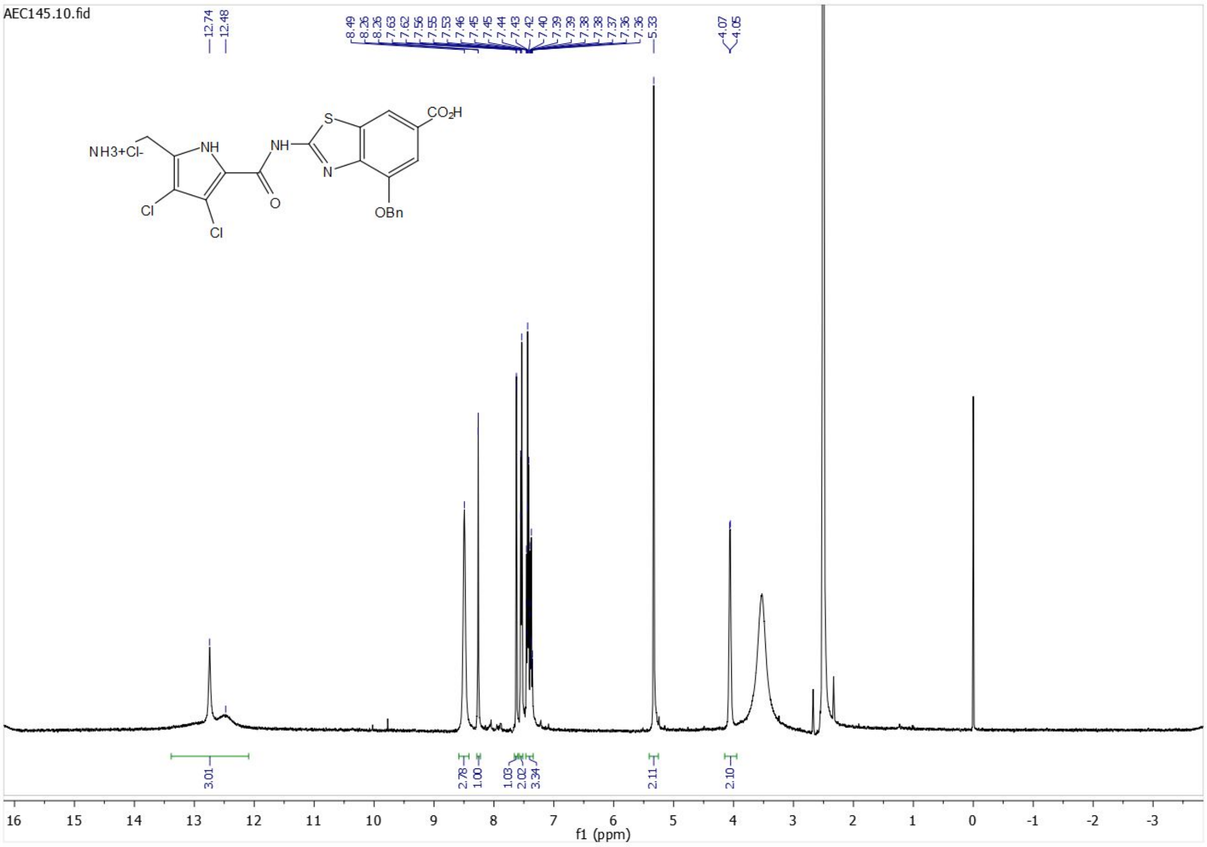

Compound **5·HCl**, ^{13}C NMR (100 MHz, DMSO-d_6):

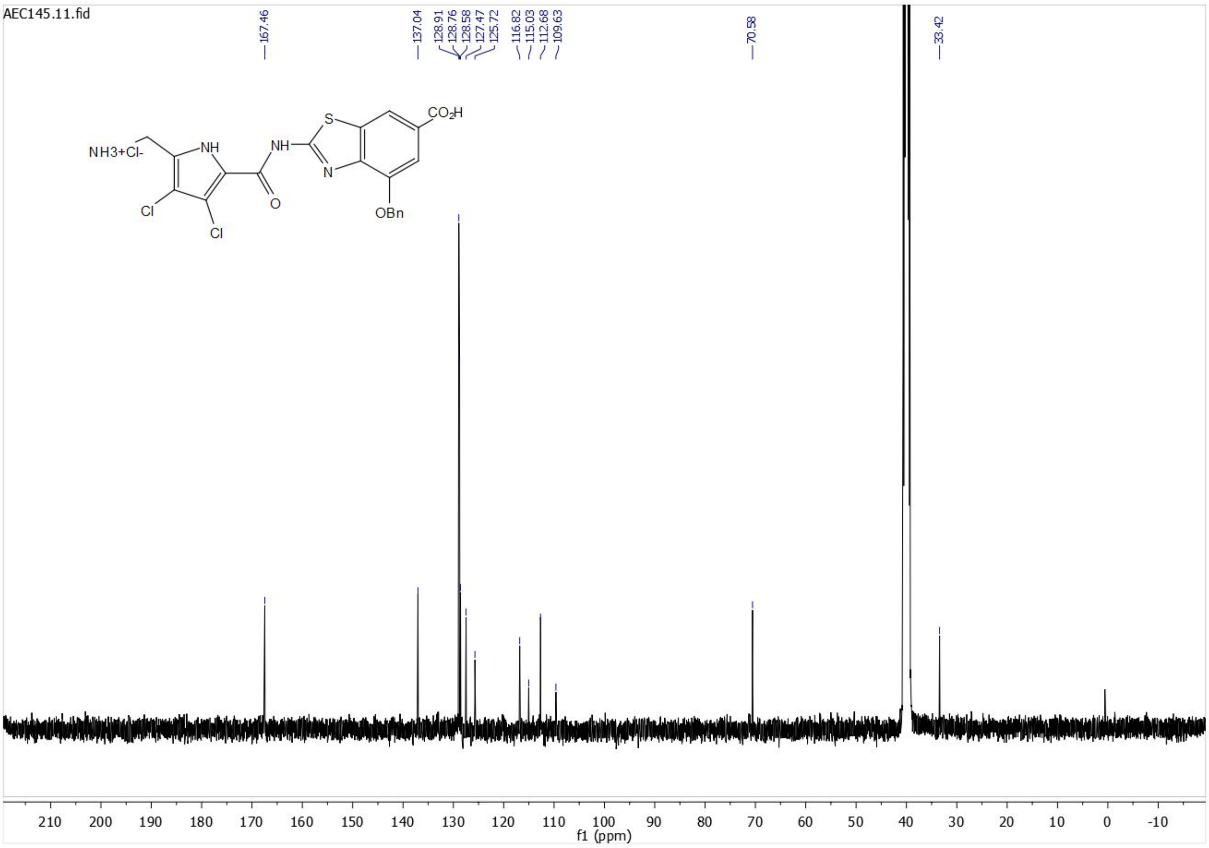

Compound **30**, ^{1}H NMR (400 MHz, DMSO-d_6):

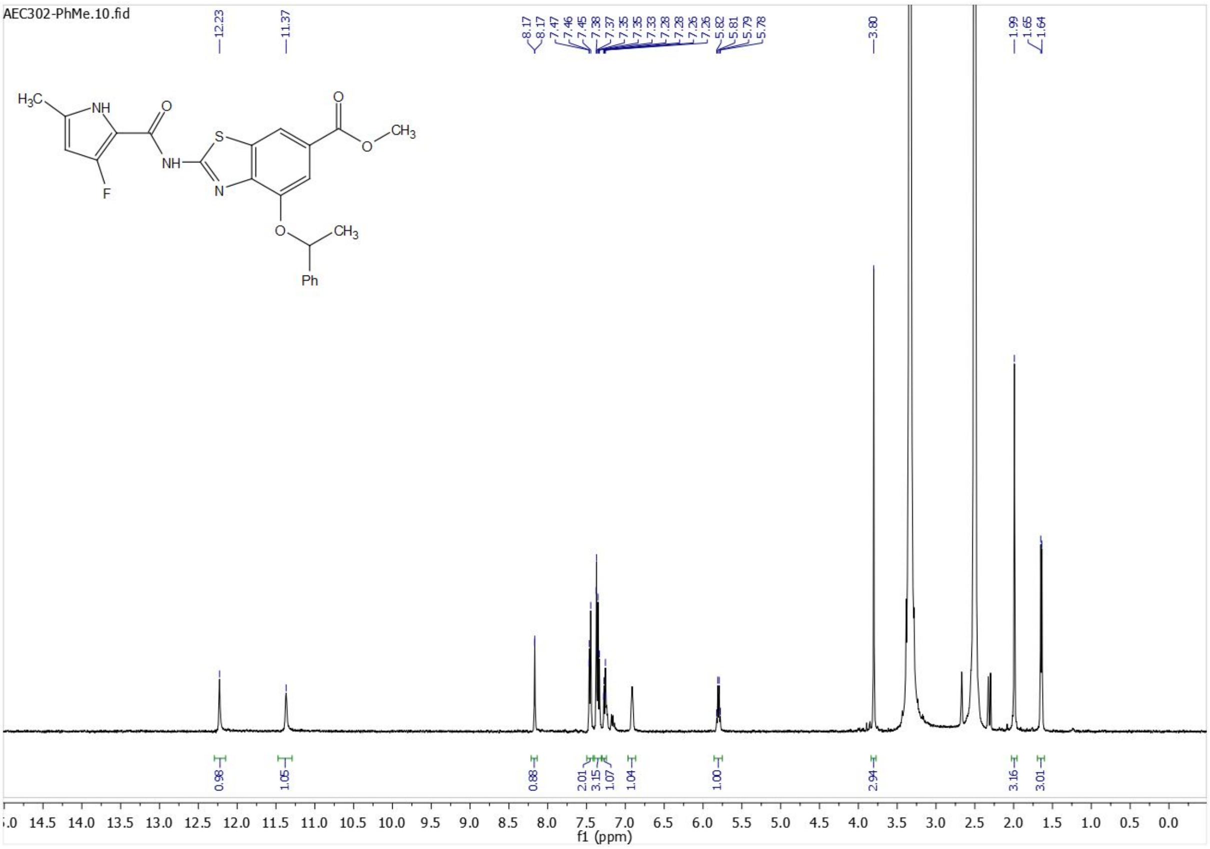

Compound **31**, ^{1}H NMR (400 MHz, DMSO-d_6):

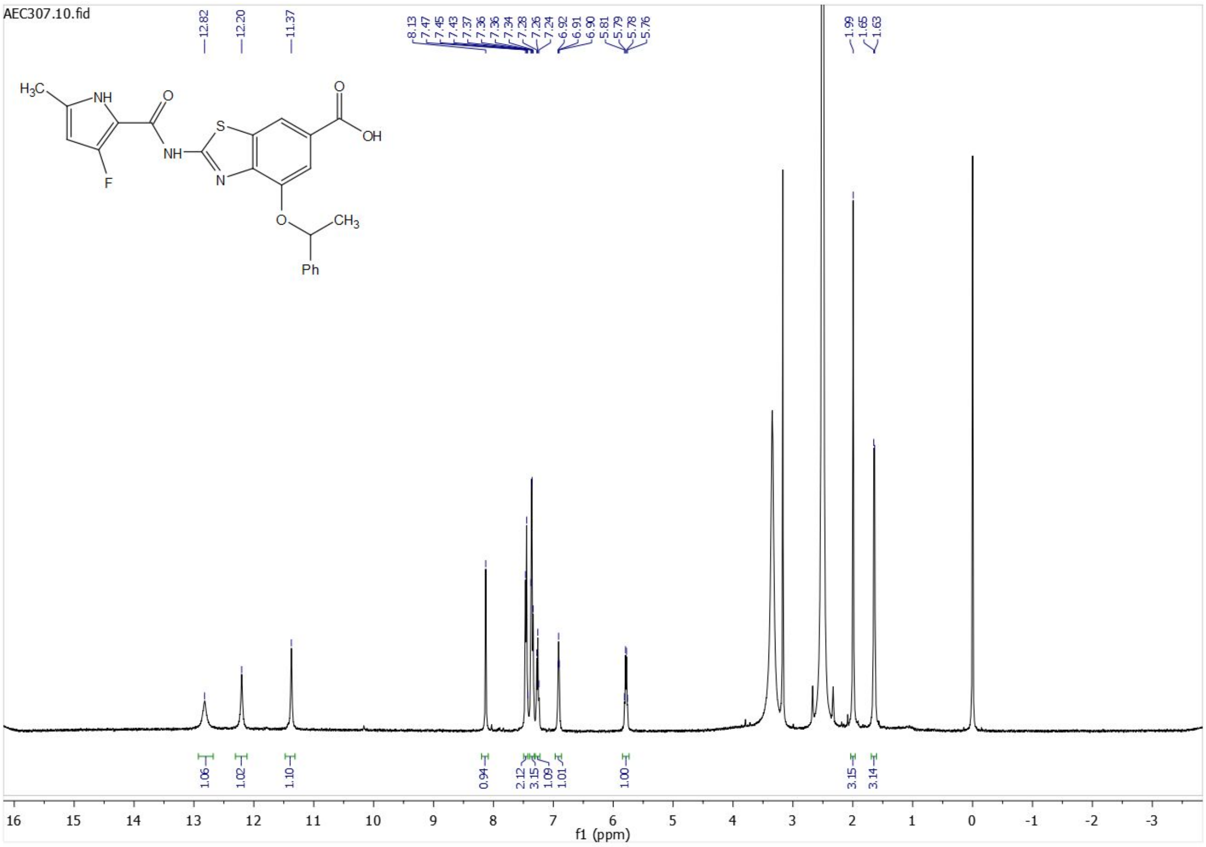

Compound **31**, ^{13}C NMR (100 MHz, DMSO-d_6):

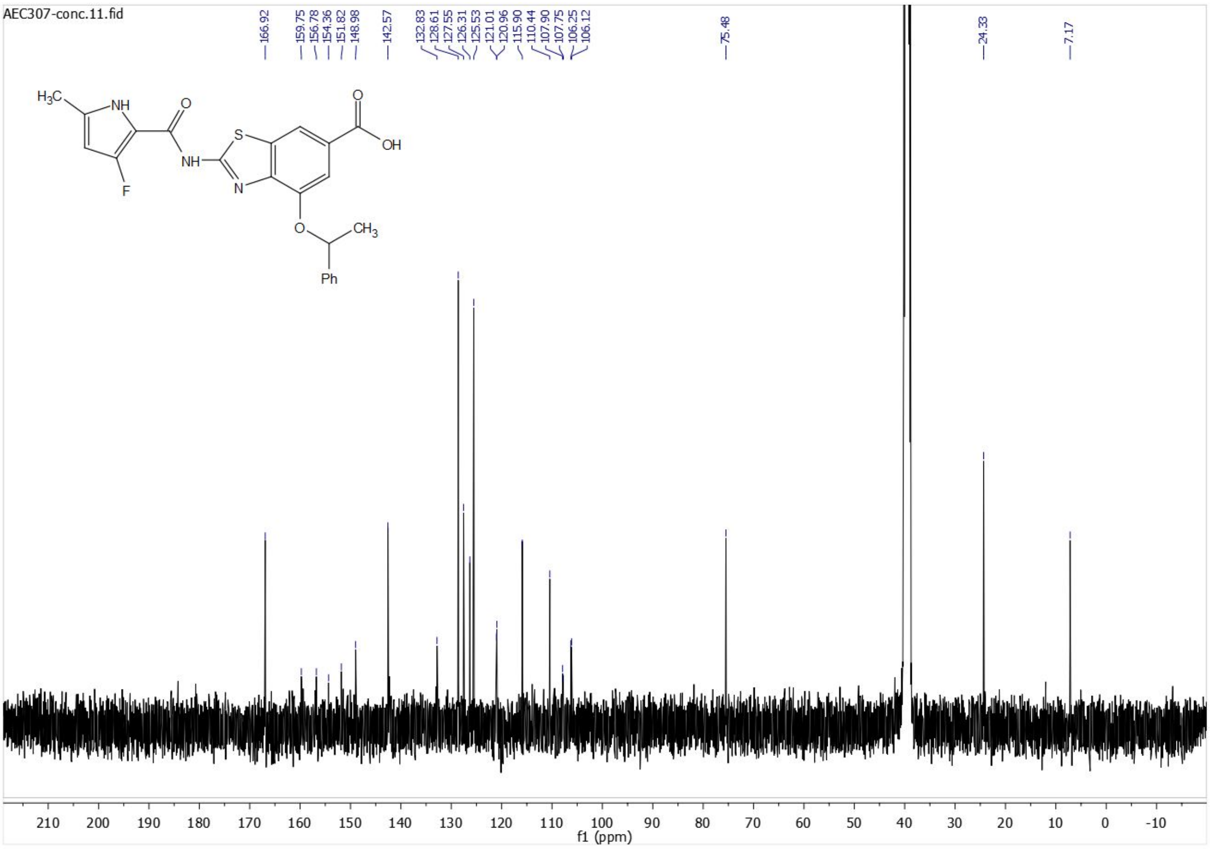

Compound **32**, ^{1}H NMR (400 MHz, DMSO-d_6):

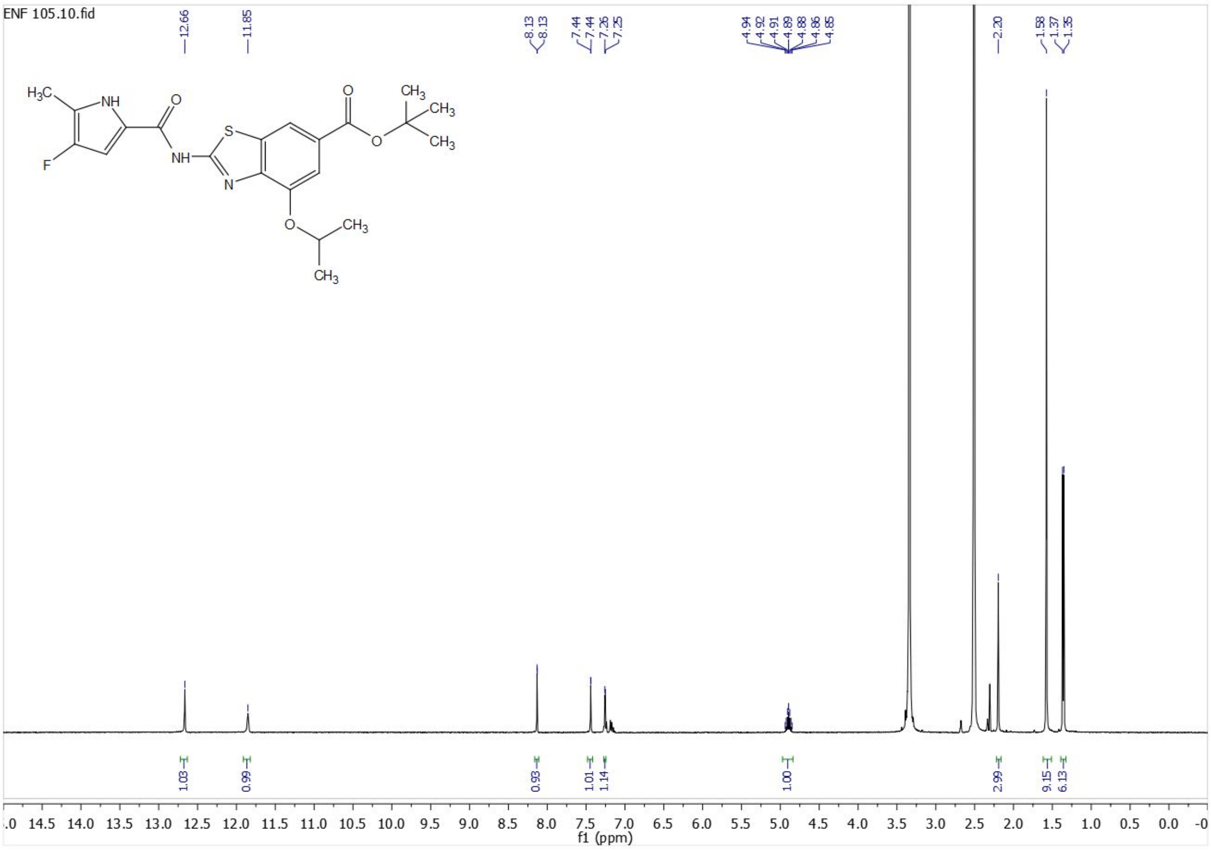

Compound **33**, ^{1}H NMR (400 MHz, DMSO-d_6):

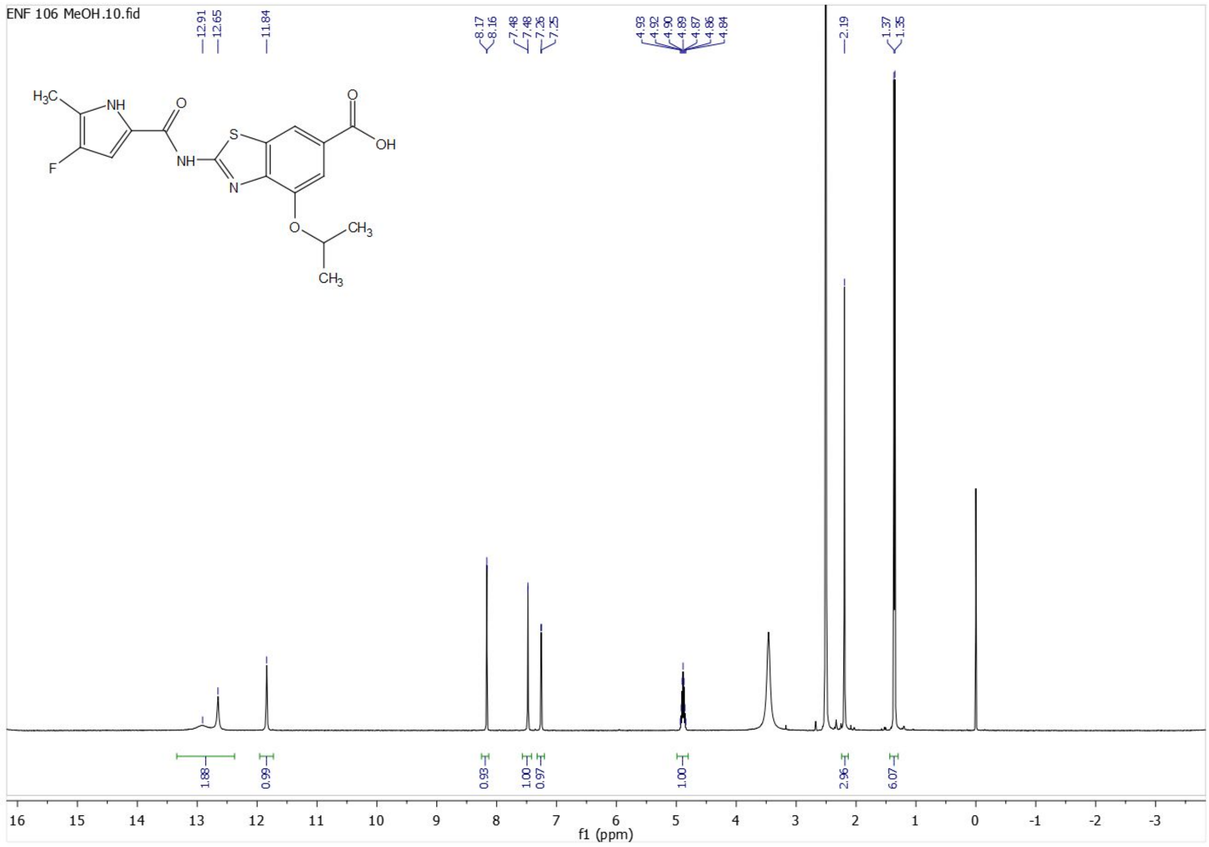

Single crystal X-ray diffraction analysis

The crystals were placed in oil, and a single crystal was selected, mounted on a glass fibre and placed in a low-temperature N_2 stream.

X-ray diffraction data collection was carried out on a Bruker PHOTON III DUO CPAD diffractometer equipped with an Oxford Cryosystem liquid N_2 device, using Mo-Kα radiation (λ = 0.71073 Å). The crystal-detector distance was 37 mm. The cell parameters were determined (APEX3 software) [1] from reflections taken from 1 set of 180 frames at 1 s exposure. The structures were solved using the program SHELXT-2014 [2]. The refinement and all further calculations were carried out using SHELXL-2014 [3]. The hydrogen atoms of the NH groups were located from Fourier difference. The other H-atoms were included in calculated positions and treated as riding atoms using SHELXL default parameters. The non-H atoms were refined anisotropically, using weighted full-matrix least-squares on F^2. A semi-empirical absorption correction was applied using SADABS in APEX3 [1]; transmission factors: T_{min}/T_{max} = 0.6896/0.7460 and T_{min}/T_{max} = 0.6867/0.7460 for **10** and **11** respectively.

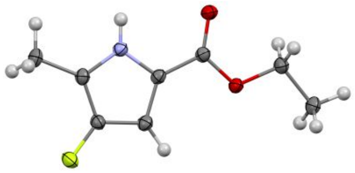

Figure S1. Single crystal X-ray analysis of **10**. One of two chemically identical molecules in the asymmetric unit is shown. Thermal ellipsoids are drawn at the 50% probability level and hydrogen atoms are depicted as spheres of arbitrary radius. Pertinent crystallographic data: triclinic crystal system, *P*-1 space group (No. 2), *a* = 7.9582(11) Å, *b* = 9.7232(13) Å, *c* = 11.9573(15) Å, *α* = 69.282(5)°, *β* = 78.205(5)°, *γ* = 74.208(50)° , *V* = 826.72(19) Å^3, *Z* = 2, *Z′* = 2, *T* = 120(2) K, *μ* = 0.11 mm^{-1}.

CCDC number: 2022199

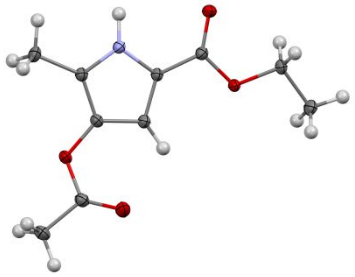

Figure S2. Single crystal X-ray analysis of **11**. One of two chemically identical molecules in the asymmetric unit is shown. Thermal ellipsoids are drawn at the 50% probability level and hydrogen atoms are depicted as spheres of arbitrary radius. Pertinent crystallographic data: monoclinic crystal system, $P2_1/c$ space group (No. 14), *a* = 12.3507(5) Å, *b* = 13.6579(2) Å, *c* = 12.1808(5) Å, *β* = 90.577(2)°, *V* = 2054.61(15) Å^3, *Z* = 2, *Z′* = 2, *T* = 120(2) K, *μ* = 0.11 mm^{-1}.

CCDC number: 2022198

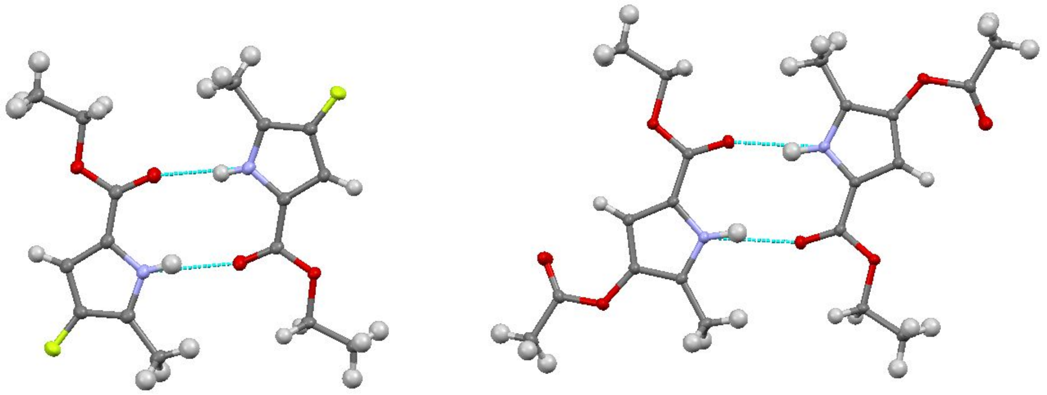

Figure S3. Hydrogen-bonded dimers of **10** (left) and **11** (right): tandem hydrogen bonds between pyrrole NH and carbonyl oxygen (graph set notation $R_2^2(10)$) connect two crystallographically independent molecules in dimers in both structures. Donor–acceptor distances are 2.817(3) and 2.820(3) Å in **10**; 2.7877(13) and 2.8156(13) Å in **11**. Independent molecules are approximately related by non-crystallographic two-fold rotation axis in both structures

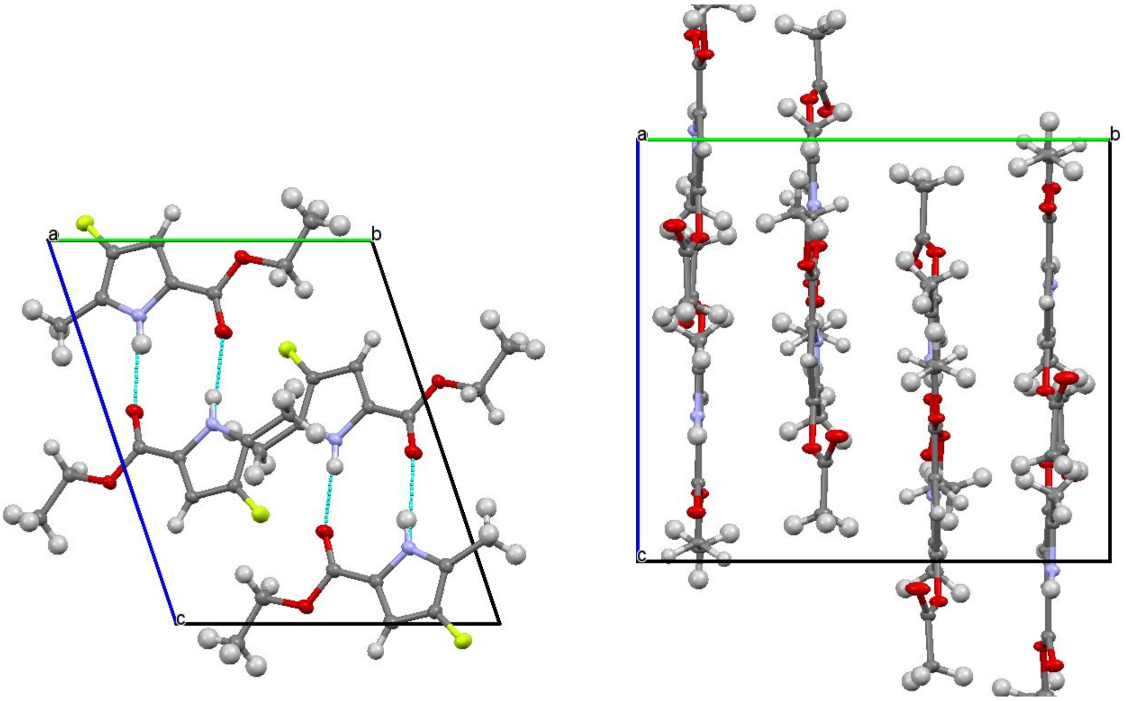

Figure S4. Packing of molecules **10** (left) and **11** (right): a view along a axis. Hydrogen bonded dimers are arranged in sheets parallel to (111) crystallographic plane in **10**, and (010) crystallographic plane in **11**.

References

[1] "M86-EXX229V1 APEX3 User Manual", Bruker AXS Inc., Madison, USA, 2016.

[2] Sheldrick, G. M. SHELXT - Integrated space-group and crystal-structure determination. *Acta Cryst. A* **2015***, 71*, 3-8.

[3] Sheldrick, G. M. Crystal structure refinement with SHELXL. *Acta Cryst. C* **2015**, *71*, 3-8.
