# Supplementary material for: Practical Synthesis and Application of Halogen-Doped Pyrrole Building Blocks
Source: ACS Omega. 2021 Mar 30;6(14):9723–30. doi: 10.1021/acsomega.1c00331 (PMC8047689; doi:10.1021/acsomega.1c00331)

checkCIF/PLATON report

Structure factors have been supplied for datablock(s) flik190208

THIS REPORT IS FOR GUIDANCE ONLY. IF USED AS PART OF A REVIEW PROCEDURE FOR PUBLICATION, IT SHOULD NOT REPLACE THE EXPERTISE OF AN EXPERIENCED CRYSTALLOGRAPHIC REFEREE.

No syntax errors found.   CIF dictionary   Interpreting this report

Datablock: flik190208

```
Bond precision:  C-C = 0.0017 A          Wavelength=0.71073

Cell:            a=12.3507(5)     b=13.6579(6)       c=12.1808(5)
                 alpha=90         beta=90.577(2)     gamma=90
Temperature:     120 K

                Calculated                  Reported
Volume          2054.61(15)                 2054.61(15)
Space group     P 21/c                      P 21/c
Hall group      -P 2ybc                     -P 2ybc
Moiety formula  C10 H13 N O4                C10 H13 N O4
Sum formula     C10 H13 N O4                C10 H13 N O4
Mr              211.21                      211.21
Dx,g cm-3       1.366                       1.366
Z               8                           8
Mu (mm-1)       0.106                       0.106
F000            896.0                       896.0
F000'           896.52
h,k,lmax        17,19,17                    17,19,17
Nref            6024                        6002
Tmin,Tmax       0.963,0.974                 0.687,0.746
Tmin'           0.958

Correction method= # Reported T Limits: Tmin=0.687 Tmax=0.746
AbsCorr = MULTI-SCAN

Data completeness= 0.996          Theta(max)= 30.054

R(reflections)= 0.0509( 5474)     wR2(reflections)= 0.1441( 6002)

S = 1.120                    Npar= 285
```

The following ALERTS were generated. Each ALERT has the format
test-name_ALERT_alert-type_alert-level.
Click on the hyperlinks for more details of the test.

Alert level B

```
PLAT934_ALERT_3_B Number of (Iobs-Icalc)/Sigma(W) > 10 Outliers ..          2 Check
```

Alert level C

```
PLAT906_ALERT_3_C Large K Value in the Analysis of Variance ......      4.365 Check
PLAT911_ALERT_3_C Missing FCF Refl Between Thmin & STh/L=    0.600         19 Report
PLAT913_ALERT_3_C Missing # of Very Strong Reflections in FCF ....          6 Note
PLAT977_ALERT_2_C Check Negative Difference Density on H5A              -0.35 eA-3
PLAT977_ALERT_2_C Check Negative Difference Density on H5C              -0.39 eA-3
```

Alert level G

```
PLAT910_ALERT_3_G Missing # of FCF Reflection(s) Below Theta(Min).          1 Note
PLAT912_ALERT_4_G Missing # of FCF Reflections Above STh/L=  0.600          3 Note
PLAT978_ALERT_2_G Number C-C Bonds with Positive Residual Density.         13 Info
PLAT992_ALERT_5_G Repd & Actual _reflns_number_gt Values Differ by          2 Check
```

```
   0 ALERT level A = Most likely a serious problem - resolve or explain
   1 ALERT level B = A potentially serious problem, consider carefully
   5 ALERT level C = Check. Ensure it is not caused by an omission or oversight
   4 ALERT level G = General information/check it is not something unexpected

   0 ALERT type 1 CIF construction/syntax error, inconsistent or missing data
   3 ALERT type 2 Indicator that the structure model may be wrong or deficient
   5 ALERT type 3 Indicator that the structure quality may be low
   1 ALERT type 4 Improvement, methodology, query or suggestion
   1 ALERT type 5 Informative message, check
```

Validation response form

Please find below a validation response form (VRF) that can be filled in and pasted into your CIF.

```
# start Validation Reply Form
_vrf_PLAT934_flik190208
;
PROBLEM: Number of (Iobs-Icalc)/Sigma(W) > 10 Outliers ..          2 Check
RESPONSE: ...
;
_vrf_PLAT906_flik190208
;
PROBLEM: Large K Value in the Analysis of Variance ......      4.365 Check
RESPONSE: ...
;
_vrf_PLAT911_flik190208
;
PROBLEM: Missing FCF Refl Between Thmin & STh/L=    0.600         19 Report
RESPONSE: ...
;
_vrf_PLAT913_flik190208
;
PROBLEM: Missing # of Very Strong Reflections in FCF ....          6 Note
RESPONSE: ...
;
_vrf_PLAT977_flik190208
;
```

```
PROBLEM: Check Negative Difference Density on H5A             -0.35 eA-3
RESPONSE: ...
;
# end Validation Reply Form
```

It is advisable to attempt to resolve as many as possible of the alerts in all categories. Often the minor alerts point to easily fixed oversights, errors and omissions in your CIF or refinement strategy, so attention to these fine details can be worthwhile. In order to resolve some of the more serious problems it may be necessary to carry out additional measurements or structure refinements. However, the purpose of your study may justify the reported deviations and the more serious of these should normally be commented upon in the discussion or experimental section of a paper or in the "special_details" fields of the CIF. checkCIF was carefully designed to identify outliers and unusual parameters, but every test has its limitations and alerts that are not important in a particular case may appear. Conversely, the absence of alerts does not guarantee there are no aspects of the results needing attention. It is up to the individual to critically assess their own results and, if necessary, seek expert advice.

Publication of your CIF in IUCr journals

A basic structural check has been run on your CIF. These basic checks will be run on all CIFs submitted for publication in IUCr journals (*Acta Crystallographica, Journal of Applied Crystallography, Journal of Synchrotron Radiation*); however, if you intend to submit to *Acta Crystallographica Section C* or *E* or *IUCrData*, you should make sure that full publication checks are run on the final version of your CIF prior to submission.

Publication of your CIF in other journals

Please refer to the *Notes for Authors* of the relevant journal for any special instructions relating to CIF submission.

PLATON version of 05/12/2020; check.def file version of 05/12/2020

Datablock flik190208 - ellipsoid plot

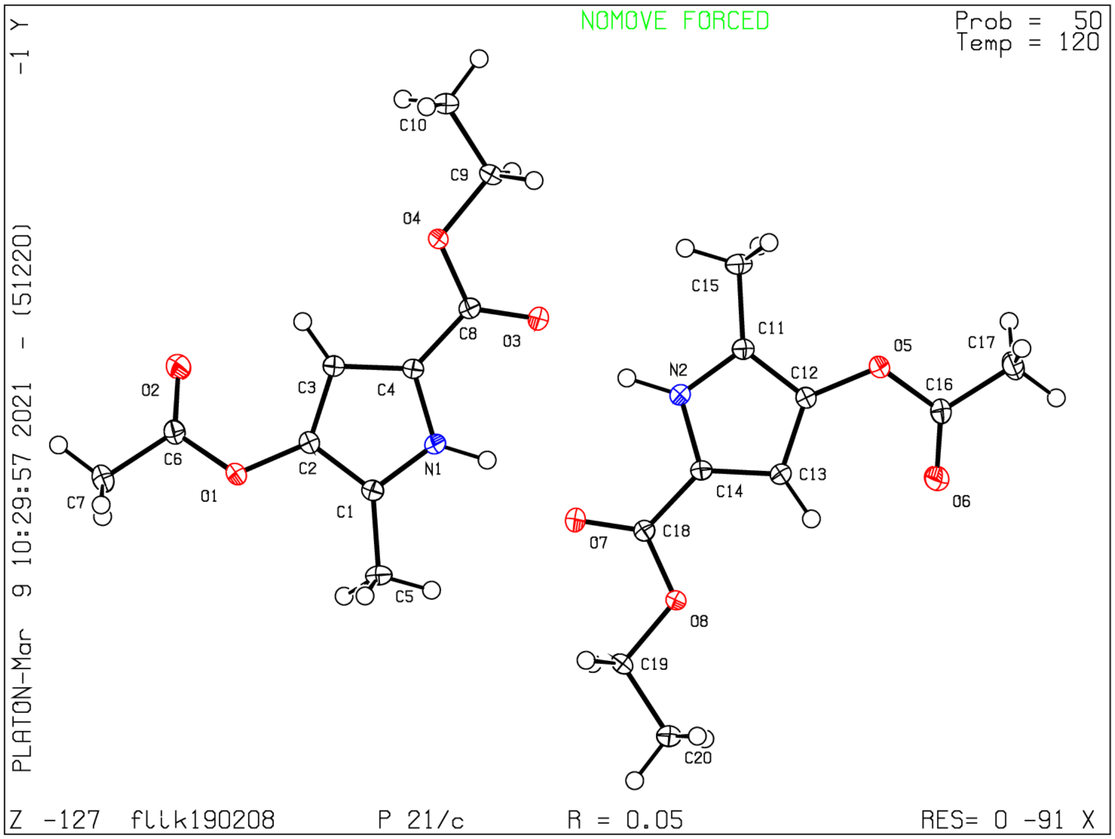

Supplement: Supplementary file 4 — ao1c00331_si_004.pdf [file ao1c00331_si_004.pdf]
